# Graphene oxide assisted light-up aptamer selection against Thioflavin T for label-free detection of microRNA

**DOI:** 10.1038/s41598-021-83640-z

**Published:** 2021-02-22

**Authors:** Md Mamunul Islam, Victoria Michele Ghielmetti, Peter B. Allen

**Affiliations:** Department of Chemistry, 001 Renfrew Hall, University of Idaho, 875 Perimeter Dr, Moscow, ID 83844-2343 USA

**Keywords:** Analytical chemistry, DNA, Enzyme mechanisms, Cancer, Chemistry, Nanoscience and technology

## Abstract

We selected an aptamer against a fluorogenic dye called Thioflavin T (ThT). Aptamers are single-stranded DNA that can bind a specific target. We selected the ThT aptamer using graphene oxide assisted SELEX and a low-cost Open qPCR instrument. We optimized, minimized, and characterized the best aptamer candidate against ThT. The aptamer, ThT dye, and the enzymatic strand displacement amplification (SDA) were used in a label-free approach to detect the micro RNA miR-215 in saliva and serum. The aptamer confers higher specificity than intercalating dyes but without expensive covalently modified DNA probes. This isothermal, low-cost, simple method can detect both DNA and RNA. The target, miR-215, was detected with a limit of detection of 2.6 nM.

## Introduction

We developed an aptamer to report the activity of an isothermal, enzymatic amplification reaction called Strand Displacement Amplification (SDA). The aptamer binds a commercially available small molecule dye called Thioflavin T (ThT). ThT is known to be a light-up probe in the presence of many DNA structures, including several G-quadruplexes (G4)^1,2^, G-triplex^3^, bulged, and mismatched structures^4,5^. Due to its easy availability, water solubility, and convenient excitation and emission wavelength, ThT has been used in many biosensing applications (e.g., monitoring RNA metabolism^6^, RNA G-quadruplexes^7^, catalytic DNA amplifier^8^, and CRISPR/Cas9n system^9^).

We selected a new aptamer using graphene oxide (GO) assisted SELEX, or GO-SELEX^10,11^ (systematic evolution of ligands by exponential enrichment), a process that selects DNA based on its ability to change from single-stranded to higher structured upon addition of a target molecule (i.e., ThT). G4 structures bind to ThT and induce fluorescence, but structures like G4 are not ideal reporters for reactions like SDA. The G4/ThT complex has a high melting temperature. These stable structures can interfere with desired enzymatic and non-enzymatic reactions. It is also known that G4 can bind with other species in solution (e.g., metal ions^12^, hemin^13^), which could make for less predictable behavior in the system. We sought an easily denatured, dynamic reporter system that would respond only to a specific analyte.

We obtained such an aptamer with Graphene oxide-SELEX. GO-SELEX was especially well-suited to this application because we could select against strongly structured DNA. GO selectively binds to ssDNA. After binding, the GO-associated ssDNA can be separated by centrifugation. We used this property to collect the bound, unstructured DNA (in the pellet) or the unbound, structured DNA (in the supernatant). We applied successive rounds of negative and positive selection. We performed negative selection by removing the supernatant containing the structured DNA in the absence of ThT (retaining the ssDNA on the GO pellet). For positive selection, we added the ThT to the mix and collected the structured DNA (retaining the supernatant). We recovered sequence data with next-generation sequencing. After evaluation and minimization, we characterized a DNA aptamer that binds to ThT and induces fluorescence, but that has no predicted structure by NUPACK^14^. The aptamer has a G-score^15^ of 10, which is below the range of scores for a typical G4 structure^16^. The aptamer sequence also has a G4Hscore^17^ of 0.833, which is lower than 95% of sampled G4 structures^18^. To date, there is only one other group that has selected an aptamer against this target^19^. However, our aptamer generates three times more intense fluorescence when it binds to ThT.

We applied our new aptamer to detect a strand displacement amplification (SDA) reaction. SDA is an isothermal and enzymatic amplification. It uses a displacing polymerase and a nicking endonuclease to make many single-stranded copies of a template oligonucleotide^20^. Briefly, a template DNA molecule is designed with a nicking endonuclease recognition site. The nicking endonuclease generates a break in the phosphodiester backbone of one strand. The strand displacing polymerase recognizes the 3′ end of the nicked strand and initiates polymerization. The polymerization process displaces the existing ssDNA into the solution. The process then repeats. SDA based detection has been previously reported as a detector of nucleic acids like miRNA^21^.

SDA can be reported by many techniques. It has previously been detected with dyes like SYBR Green II^22^. However, such dyes are nonspecific and interact with any amplicon, including undesired side products. Molecular beacons^23^ can report SDA and are specific, but molecular beacons introduce high cost and high sensitivity to degradation. Degraded molecular beacons will spontaneously de-quench and generate background fluorescence.

We used a simple SDA method to produce many copies of our aptamer. The SDA-generated aptamers induced fluorescence of Thioflavin-T (ThT). The aptamer yields a specific signal for the desired SDA product without the additional expense or purity requirements associated with dye-modified oligonucleotides.

We optimized the reaction conditions for SDA with ThT aptamer detection, which allowed us to detect miRNA-215 (a short oligonucleotide sequence of 21 bases) at low nanomolar concentrations. It has an essential regulatory role for tumor suppressor gene p53^24^. The biological importance of miR-215 has been reported as an essential agent in different cellular processes^24–27^. miR-215 is an important biomarker for colon cancer that has been reported previously^25,26,28^. Irregular expression of miR-215 was reported to be related to other cancers, too. Levels of miR-215 are related to myeloid leukemia^24^, nephroblastoma^29^, and breast cancer^30^. Therefore, it is crucial to develop a sensitive detection method of miR-215. RT-PCR is currently used as a quantification method of miR-215. However, RT-PCR is limited in application in many places due to its non-isothermal nature. SDA is a simple, isothermal method, which does not require reverse transcriptase enzyme to detect miRNA.

In this study, we have developed an SDA method to specifically detect miR-215. This method has several advantages. It is label-free, low-cost, simple, room temperature, isothermal (no thermocycling needed), specific, and can potentially detect miRNAs in biological samples. We propose that this method can be used for the myriad polymerase-endonuclease-systems. Such systems have been demonstrated as linear amplifiers^31^, exponential amplifiers^21^, and complex reaction networks^32^.

## Methods

### Materials

DNA and RNA oligonucleotides were purchased from Integrated DNA Technologies, IDT (Coralville, IA, USA). Nuclease-free water and SYBR Green II were purchased from Thermo Scientific (Waltham, MA, USA). Sodium chloride (NaCl) was obtained from EMD Chemicals (Gibbstown, Germany). Potassium chloride (KCl) was obtained from Avantor Performance Materials, PA, USA.Tris base (molecular grade) was purchased from Promega Corporation (Madison WI, USA). Sodium tetraborate decahydrate was purchased from Sigma-Aldrich (St. Louis, MO, USA). ThT was purchased from Arcos Organics, NJ, USA. Graphene oxide was purchased from ACS material (Pasadena, CA, USA). Ethanol was purchased from AAPER Alcohol & Chemical Company (Shelbyville, KY, USA). Sodium acetate and butanol were purchased from Fisher Scientific (NJ, USA). PCR master mix was purchased from Thermo Scientific (Waltham, MA, USA). Gel loading dye, *Bst 2.0* DNA Polymerase, dNTPs Solution Mix, Nt.BstNBI and Isothermal amplification buffer pack were purchased from New England Biolabs (Ipswich, MA, USA). Agarose was purchased from Life Technologies (Carlsbad CA, USA). Acrylamide/bis-Acrylamide was purchased from Research Product International (Mount Prospect, IL, USA). EvaGreen and GelRed were purchased from Biotium (Hayward, CA, USA). All reagents used were of analytical grade. Well plates (384, black with clear bottoms) were purchased from Corning (Glendale, AZ, USA). Fetal bovine serum was obtained from Atlas biologicals (Fort Collins, CO 80524, USA).

### Aptamer selection

The thioflavin T (ThT) aptamer was selected through eight rounds of the selection process. Round 1 of the selection processes began by mixing equimolar quantities of ThT and the DNA pool (E7N40 synthetic DNA pool, based on a published design^33^, specified in Table S1 of the supplementary information) in selection buffer (50 mM Tris, 150 mM NaCl, pH 7.4) and allowing it to incubate while tumbling for 1 h. Then 5 mg/mL of graphene oxide (GO) was added to this mixture, and the solution was allowed to incubate while tumbling for another 2 h. The GO was then removed from the solution by centrifuging the sample for 15 min and extracting the supernatant. The supernatant was then filtered with a 0.45 µm spin filter to remove any remaining GO particles. The supernatant was concentrated using the standard butanol concentration protocol until the supernatant had a final volume of 100 µL. The sample was then mixed with sodium acetate buffer (3 M Sodium Acetate, pH 5.2), 250 µL ethanol, and 2 µL of glycogen and placed in a freezer at -20 °C for 30 min. The solution was then centrifuged at 4 °C for another 30 min, at which point a white pellet was visible. The liquid was removed from the centrifuge tube, and the pellet dried at 37 °C for 2 h. The pellet was resuspended in 50 µL of water (boiled Millipore H_2_O bottled in sterilized containers). This solution went through an 8-cycle PCR cycle-course by mixing the solution with 2 × Taq PCR Master Mix, P1, and P2 (two primers designed for the E7N40 DNA pool specified in Table S1 of the supplementary information). A cycle was defined as 95 °C for 15 s, 59 °C for 15 s, and 69 °C for 30 s. A portion of the amplified solution was mixed with a qPCR mixture (2 × Taq PCR Master Mix, the two primers, and 20 × EvaGreen) and placed in an Open qPCR (Chai Biotechnologies, Santa Clara, CA) that monitored the fluorescence of the amplified sample over time.

Simultaneously, three control samples of E7N40 DNA (100 nM, 10 nM, and 1 nM) and water were mixed with the qPCR mixture and were run alongside the amplified sample. The Open qPCR was run with the same cycle settings as the 8-course PCR cycle-course. Once completed, the remaining sample was amplified using PCR with the same cycle settings. The sample was mixed with a PCR mixture consisting of 2 × Taq PCR Master Mix, fluorescein modified P1 (P1-f), and acrydite modified P2 (P2-Acr) (modified primers specified in Table S1) and was run for the number of cycles determined by Open qPCR. A small portion of the PCR product was mixed with a loading dye and placed in the wells of a 1% Agarose Gel along with a ladder and a 72 base pair control sample (both mixed with the loading dye). The remaining PCR product was denatured in a 5% PAGE gel (7 M, urea) following standard protocol. The product from the PAGE gel was concentrated using the standard butanol concentration procedure. The DNA was then precipitated out of the solution using ethanol and sodium acetate as before. The Round 1 DNA pool was finally quantified with Open qPCR.

Round 2 was similar to Round 1 with two key differences. The first difference is that the Round 1 DNA pool was used instead of the E7N40 DNA pool at the beginning. The second difference is that after the supernatant was collected from the DNA-GO complex, the supernatant was not concentrated with butanol or precipitated with ethanol and sodium acetate. The supernatant also did not go through an initial 8-cycle PCR cycle-course. After the supernatant was collected, it was then analyzed by Open qPCR and continued the Round 1 protocol from there.

In Round 3, the negative selection was introduced to the protocol. The Round 2 DNA pool was first mixed with GO and allowed to incubate at room temperature for 30 min. The supernatant was extracted using a centrifuge at room temperature and discarded. A sample of ThT equimolar to the original Round 2 DNA pool was added to the remaining DNA-GO complex. This solution was then allowed to incubate overnight while rotating, and an extra selection buffer was added to facilitate better rotation. The solution was centrifuged, and the supernatant was collected. This eluted DNA was analyzed with Open qPCR and continued the Round 2 protocol from there. Round 4 through Round 8 followed the protocol of Round 3 with no deviations.

### ThT aptamer characterization

Based on their higher relative abundance, five aptamer candidates (Apt1, Apt2, Apt3, Apt4, Apt5, (sequences are given in Table 1) were selected from the Round 8 product. Using spectramax ID3 plate reader, the fluorescence intensity at 510 nm of these aptamer candidates was measured at 1 µM of each oligonucleotide in the presence of 5 µM of ThT dye in selection buffer as above. Further characterizations of the different fragments of the candidate Apt5 were carried out in the same way.

**Table 1:** ThT aptamer candidate sequences (1 µM aptamer candidates and 5 µM ThT in selection buffer).

Aptamer candidate	Abundance (reads)	Sequence (excluding primer binding sites)	Fluorescence enhancement
Apt1	10089 (2.3%)	TCGCGTGTGCAGAGGCGAGTAGGTGGGAGATCTGTCTGGG	9 ×
Apt2	5771 (1.31%)	GACCGGAGGGGCATCAGCTGTCCGTGAGGTTGCCGCGAG	13 ×
Apt3	4028 (0.92%)	GCACGTCCAGGACGGGGGAGCGGTGCTAGTGTCTGGCAGG	20 ×
Apt4	4223 (0.96%)	GACCGGAGGGGCATCAGCTGTCCGTGAGGTTGCCGCGAGT	14 ×
Apt5	2140 (0.49%)	GCGTAGATCGAGGCTATTAGGAGGTGGGATGCGTCAGGGC	61 ×

Images of fluorescence generated by ThT in presence of different aptamer candidates were acquired under blue light with an amber filter (FastGene Blue LED Gel Illuminator, Bulldog Bio, Portsmouth, NH).

### Circular dichroism analysis

Apt5.9-32, an arbitrary dsDNA (G-Arm /B-Br V4), ssDNA (B-Br V4), and blank samples were prepared at 10 µM in the selection buffer. A stoichiometric quantity of ssDNA and its reverse-complement were added to a solution to construct dsDNA. The solution was heated in a thermocycler to 80 °C for three minutes, followed by slow cooling at 0.1 °C per second to room temperature. All oligonucleotides were annealed before CD measurement using a Jasco 720 Circular Dichroism Spectrophotometer (Jasco, Inc., Easton, Maryland, USA).

### Binding assay of ThT and the Apt5.9-32

The binding assay of ThT and Apt5.9-32 was carried out by making six solutions with decreasing concentration (50 µM, 40 µM, 20 µM, 10 µM, 5 µM, and 2.5 µM) in a 1 µM ThT solution in the selection buffer (50 mM Tris, 150 mM NaCl, pH 7.4). A 20 µL of each solution was put in a plate reader (SpectraMax iD3) to measure their fluorescence intensities. The increase in fluorescence was taken as proportional to the Apt5.9-32/ThT complex concentration. A single binding equilibrium was assumed (ThT + Apt5.9-32 ⇌ Complex). Using Excel's solver function, a nonlinear fit was conducted. The binding assay was carried out in triplicate.

### Melt curve analysis of the Apt5.9-32

Apt5.9-32 and PW17Ext (G4) were prepared at five different concentrations (5, 2, 1, 0.5, and 0 µM) in selection buffer as above with (10 mM) or without potassium. An Open qPCR (Chai Biotechnologies, Santa Clara, CA) was used to monitor fluorescence as a function of temperature. The analysis was carried out in triplicate.

UV melt curve analysis was performed using Varian Cary 100 Bio UV–Vis Spectrophotometer. The experiment was performed in two conditions: in selection buffer as above with no ThT, and in selection buffer with 5 µM ThT solution using 1 µM of Apt5.9-32 at 260 nm. The analysis was carried out in duplicate.

### Strand displacement amplification optimization

SDA reactions at different temperatures in the range of 25–40 °C were performed. The SDA mix contained 0.04 U/μL Bst polymerase, 0.192 U/μL of nicking endonuclease, 0.24 mM dNTPs, 6 mM MgSO_4_, 1 × iso thermal buffer, and 5 µM ThT was assembled in an ice bath. Pre annealed 2.2 μL Primer/Template duplex at 1 μM was added to 19.8 μL of SDA mix. The final concentration of the Primer/Template duplex was 100 nM of the SDA temperature optimization reaction. In addition to the Primer/Template duplex, five more controls such as blank, Template, Primer only, nsTemplate only, nsSDA (Primer/nsTemplate duplex) were carried out in parallel. All oligo controls were prepared at 100 nM final concentration. Fluorescence kinetics were carried out for 1 h in a plate reader (SpectraMax iD3).

Bst polymerase was optimized at the optimum temperature of 25 °C. Seven SDA mixes were prepared in the same way as mentioned above for the seven Bst concentration points in the range of 0.01 to 0.07 U/μL. In addition to the Primer/Template duplex, three more controls, such as blank, Template, and nsSDA (Primer/nsTemplate duplex) were also carried out in parallel.

The ratio of Bst polymerase and nicking endonuclease was optimized at 25 °C in the same way. Seven different SDA mixes were assembled in ice containing seven different ratios of [Bst]: [nicking endonuclease]. The selected ratios of [Bst]: [nicking endonuclease] were 1:0.5, 1:1, 1:2, 1:4, 1:5, 1:7, 1:10. The amount of Bst polymerase was fixed at 0.04 U/μL, while the amount of nicking endonuclease was varied in the calculated ratio. In addition to the Primer/Template duplex, three more controls, such as blank, Template, and nsSDA, were also carried out in parallel.

The amount of dNTPs was optimized in the same way at 25 °C. Four different SDA mixes were prepared in ice bath containing 0.12 mM, 0.24 mM, 0.35 mM, and 0.50 mM dNTPs. The amount of Bst polymerase and nicking endonuclease was fixed at 0.04 U/μL and 0.2 U/μL, respectively. In addition to the Primer/Template duplex, three more controls, such as blank, Template, and nsSDA, were also carried out in parallel.

Mg^2+^ was optimized by performing SDA in seven different concentrations. Seven SDA mix assemblies with varying MgSO_4_ were prepared to have 2 mM, 6 mM, 8 mM, 10 mM, 15 mM, and 20 mM final concentration. The amount of Bst polymerase, nicking endonuclease, and dNTPs were fixed at 0.04 U/μL, 0.2 U/μL, and 0.24 mM, respectively. In addition to the Primer/Template duplex, three more controls, such as blank, Template, and nsSDA, were carried out in parallel.

The amount of ThT dye was optimized by making SDA mix with six different concentrations in the range of 1–20 μM while other components were kept constant. The amount of Bst polymerase, nicking endonuclease, dNTPs and Mg^2+^ was fixed at 0.04 U/μL, 0.2 U/μL, 0.24 mM, and 10 mM, respectively. In addition to the Primer/Template duplex, three more controls, such as blank, Template, and nsSDA, were carried out in parallel.

In all cases, 1 × iso thermal buffer (20 mM Tris–HCl, 10 mM (NH4)2SO4, 50 mM KCl, 2 mM MgSO4, 0.1% Tween-20, pH 8.8@25 °C) and 5 µM ThT were used. Fluorescence kinetics was carried out at 25 °C for 1 h in the plate reader (SpectraMax iD3). In all cases, oligo controls were prepared at 100 nM final concentration. Each of the above reactions for SDA optimization was carried out in triplicate at an excitation wavelength of 440 nm and an emission wavelength of 510 nm.

### SDA with different reporter system

Two SDA mixes with 0.4 × SYBR Green II and 8 μM of ThT were prepared in an ice bath. Each of the two SDA mixes contained Bst polymerase, nicking endonuclease, dNTPs, and Mg^2+^ at 0.04 U/μL, 0.2 U/μL, 0.24 mM, 10 mM, respectively. In addition to the Primer/Template duplex at 100 nM, three more controls, such as blank, Template only, and nsSDA (Primer/nsTemplate duplex), were also carried out in parallel. All oligo controls were at 100 nM final concentration. In all cases, a 1 × iso thermal buffer was used. Fluorescence kinetics was carried out at 25 °C for 1 h in the plate reader (SpectraMax iD3). Each of the reactions was carried out in triplicate.

### In-vitro detection of miR-215

Sixteen solutions of miR-215 at 0, 0.5, 1, 2, 5 10, 20, 40, 50, 60, 80, 90, 100, 150, 200, and 250 nM were made in RNAase free water. An equal volume of SDA mix and miR-215 solutions were added to all samples to make 22 μL total volume of each reaction. The concentration of the Template-215 in each reaction was constant at 100 nM. The final concentration of the SDA mix contained: 1 × iso thermal buffer, 10 mM MgSO_4_, 0.24 mM dNTPs, 0.04 U/μL Bst polymerase, 0.2 U/μL nicking endonuclease, and 8 μM ThT. Fluorescence kinetics was carried out at 25 °C for 55 min in the plate reader (SpectraMax iD3). Each reaction at all assigned concentrations of miR-215 was carried out in triplicate at an excitation wavelength of 440 nm and an emission wavelength of 510 nm. Optimization of Template-215 concentration was carried out similarly.

### SDA specificity

Nine SDA reactions were carried out in vitro using several targets at two concentration points of 50 and 20 nM. The SDA mixes were prepared as above. SDA in simulated biofluids of the same target oligonucleotides was also performed using 10% saliva (self-collected and prepared following protocols from the literature^34^) and 10% fetal bovine serum. SDA in biofluids was performed using 20 nM target oligonucleotides. The template-215 concentration was 100 nM in all SDA reactions. Fluorescence kinetics was carried out at 25 °C for 55 min in the plate reader (SpectraMax iD3). Each of the reactions was carried out in triplicate.

### PAGE analysis of SDA product

The SDA was analyzed by 12% native PAGE (including 10 mM Mg^2+^). SDA circuit reactions were carried out for 50 min at 25 °C before PAGE analysis. SDA circuit reactions and controls were assembled as follows: Lane 1, low molecular weight DNA as a ladder. Lane 2, Template-215 only. Lane 3, Primer-215 only. Lane 4, Template-215/Primer-215 duplex only. Lane 5, Apt5.9-32 only. Lane 6, Template-215 + SDA mix. Lane 7, Primer-215 + SDA mix only. Lane 8, Template-215 + SDA mix + Primer-215. The electrophoresis was carried out for 5 h at 80 V in 1 × Sodium Borate buffer (5 mM sodium borate, 10 mM MgSO_4_, pH 8). The gel was stained in 1 × GelRed (Biotium, CA, USA) for one hour. A digital fluorescence photograph was taken under blue illumination with an amber filter after staining.

## Results and Discussion

### In vitro selection

**Figure 1:**
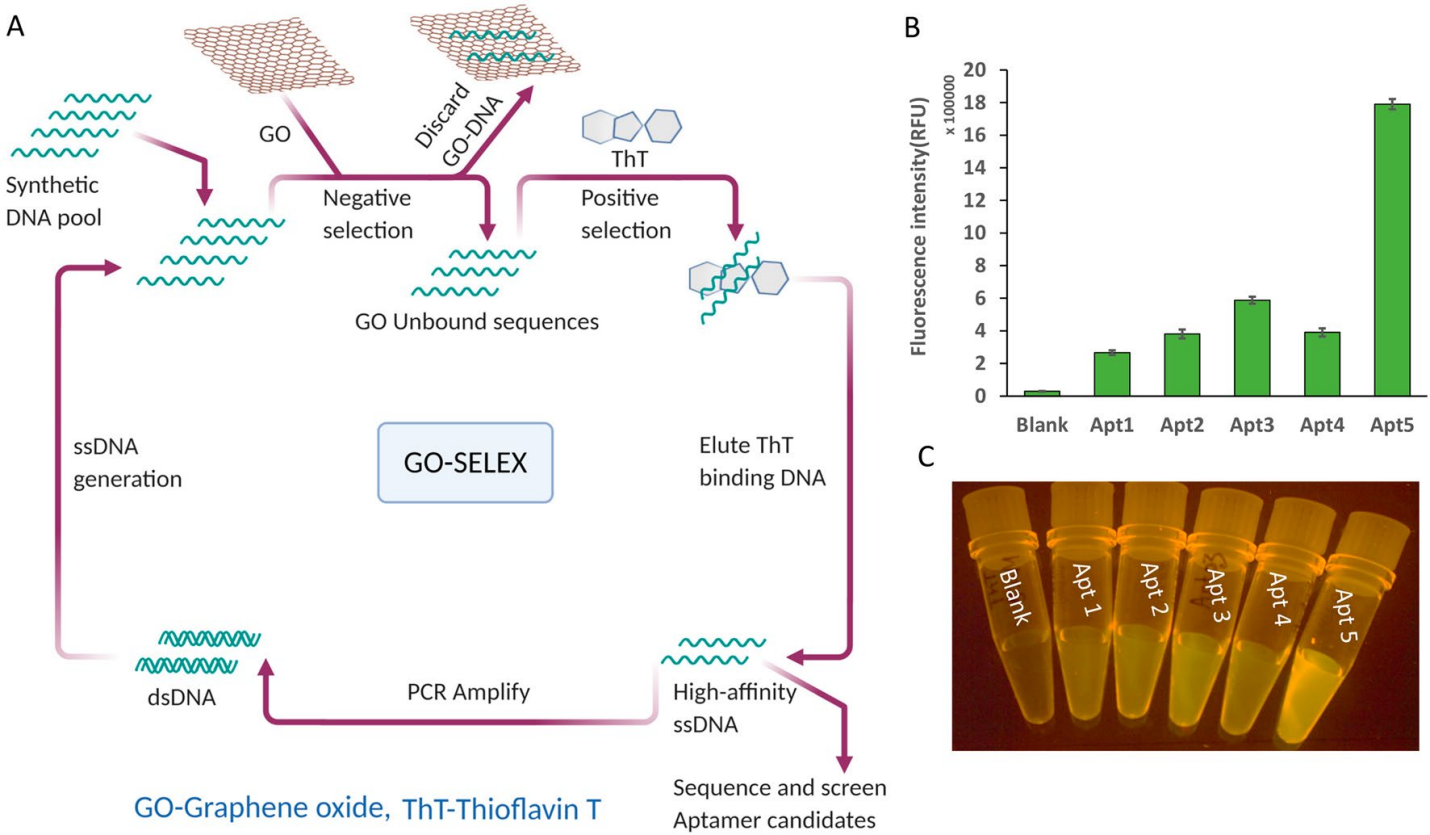
In vitro selection of ThT aptamer. (**A**) Schematic shows the process of graphene oxide-assisted in vitro selection of ThT aptamer (created with BioRender.com). (**B**) Fluorescence intensity (RFU) of five aptamer candidates and ThT only (blank). (**C**) An image of fluorescence generated by ThT in the absence and presence of five aptamer candidates selected in this study.

We performed in vitro selection to generate ThT aptamer according to an established protocol^10^. Figure 1A shows a schematic of the process. In early rounds, DNA (pool design TGCTCCGACCTTAGTCTCTG N40 GAACCGTGTAGCACAGCAGA) was mixed with ThT, and then graphene oxide (GO) was added. Unstructured, single-stranded DNA bound to GO and was discarded. Structured DNA was retained in the supernatant. The structured DNA included folded aptamers binding to ThT. Free DNA (supernatant above GO) was then amplified and regenerated using protocols developed in house and published elsewhere^35^. A negative selection step was added in later rounds: DNA was allowed to bind to GO first, and the supernatant was discarded. The DNA-coated GO was then mixed with ThT and incubated for an extended time. The DNA that became structured and eluted from the GO in the presence of ThT was retained and amplified. The in vitro selection procedure was repeated for 8 rounds.

In our selection process, we used qPCR to optimize the number of cycles for each PCR amplification step (Fig. 1A) to avoid contamination with nonspecific amplicons and increase SELEX efficiency. After the SELEX experiments, we submitted the pool to next-generation sequencing. We recovered several hundred thousand reads and ranked the recovered sequences by their abundance using in-house Python software^35^. We counted the instances of each unique sequence in the data. Most sequences were wholly unique. However, some sequences were represented many times in the data. We took these abundant sequences to be "aptamer candidates”. We synthesized the five most abundant aptamer candidate oligonucleotides (for relative abundance, see Table 1).

We denote the aptamer candidates Apt1, Apt2, Apt3, Apt4, and Apt5. Each oligonucleotide was 40 nucleotides long (Apt2 is 39 nucleotides long) and represents only the pool's random region (no primer binding sites). In the interest of generating a short aptamer, we discarded the primer motifs during the initial screening of the five aptamer candidate oligonucleotides. It is possible that some of the less fluorogenic aptamer candidates would have been functional if we had included the primer binding sites. Because our interest was in a short motif for downstream SDA applications, we chose to risk discarding longer (but possibly functional) sequences. All candidates have similar guanine base content in their sequences (varying from 17 to 19 guanine bases). Each of the five oligonucleotides can enhance ThT fluorescence (see Fig. 1B). ThT fluorescence was enhanced by a factor of 10 to 60 relative to the ThT dye by itself. The degree of fluorescence enhancement (relative to no-DNA control) is shown in Fig. 1C. Apt5, the least abundant of the five candidates, is the most fluorescent enhancer compared to the other four candidates. Apt5 generates 60 × fluorescence upon binding to ThT. Apt5 was further subjected to sequence optimization and characterization to develop a versatile reporter for the label-free strand displacement amplification (SDA) reaction.

### Aptamer characterization and optimization

**Figure 2:**
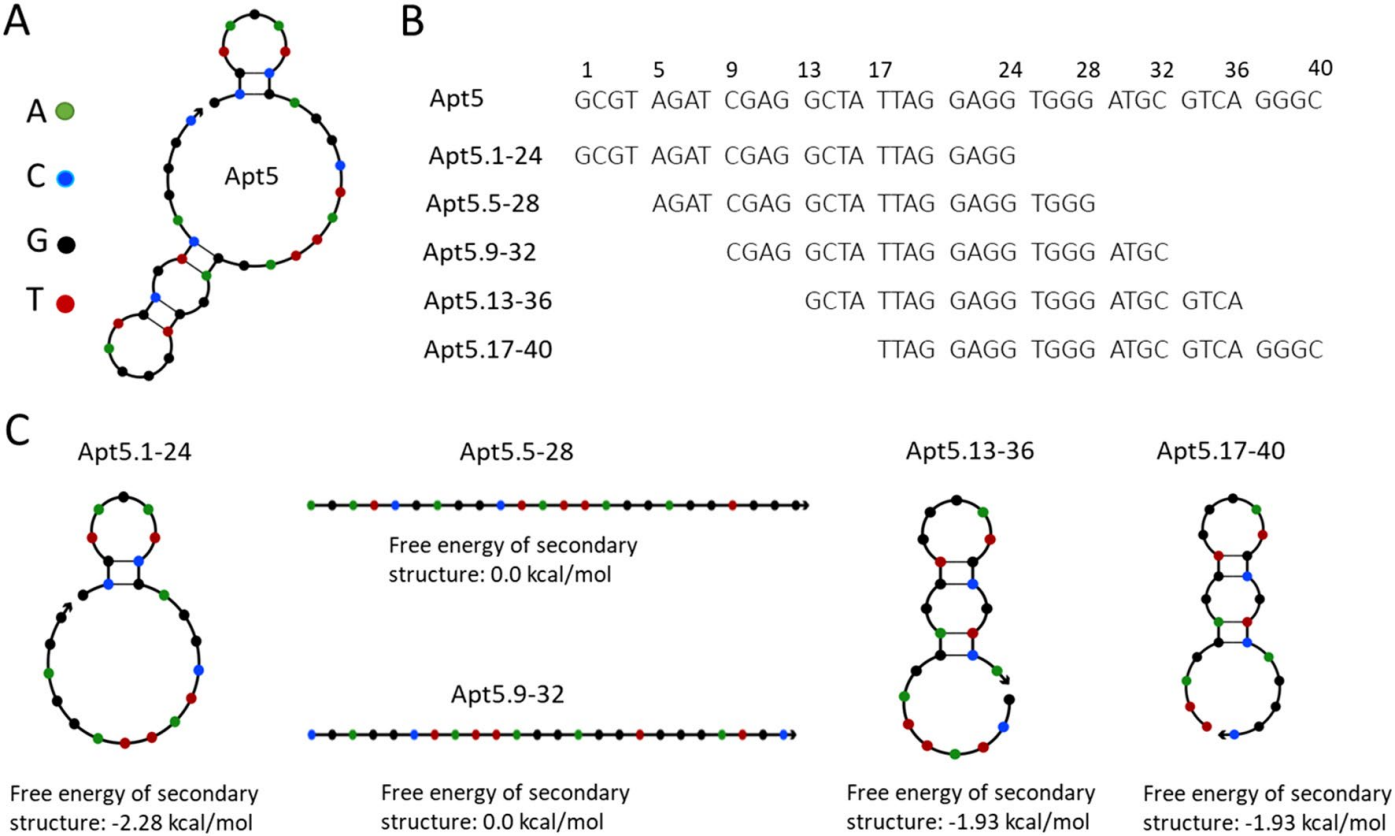
NUPACK characterization of ThT aptamer candidates^14^. (**A**) NUPACK predicted secondary structure of the Apt5. (**B**) The sequence of Apt5 and its fragments. (**C**) NUPACK predicted secondary structures of five truncated versions of Apt5.

Based on the high fluorescence enhancement of Apt5, we chose it for further study. We tested the minimum portion of the aptamer that would enhance ThT fluorescence. We inspected the secondary structure of Apt5 using NUPACK (Nucleic Acid Package), a web-based nucleic acid structural analysis tool^14^. Two hairpin structures are found in the NUPACK predicted secondary structure of Apt5 (Fig. 2A). Based on the NUPACK predicted structure of Apt5, we hypothesized that the more structured region between nucleotides 20–36 might be necessary to the ThT-binding and fluorescence enhancing activity. To test this hypothesis, we designed truncated versions of Apt5 spanning the whole Apt5 sequence. We denote these 24 nucleotide fragments by the Apt5 subsequence as follows: Apt5.1-24, Apt5.5-28, Apt5.9-32, Apt5.13-36, and Apt5.17-40 (see Fig. 2B for sequence design).

Apt5.1-24 was designed to contain the 5′ hairpin, and Apt5.17-40 includes all of the 3′ hairpin. NUPACK predicts no secondary structure for Apt5.5-28 or Apt5.9-32. All of the NUPACK structures of the Apt5 fragments are shown in Fig. 2C.

**Figure 3:**
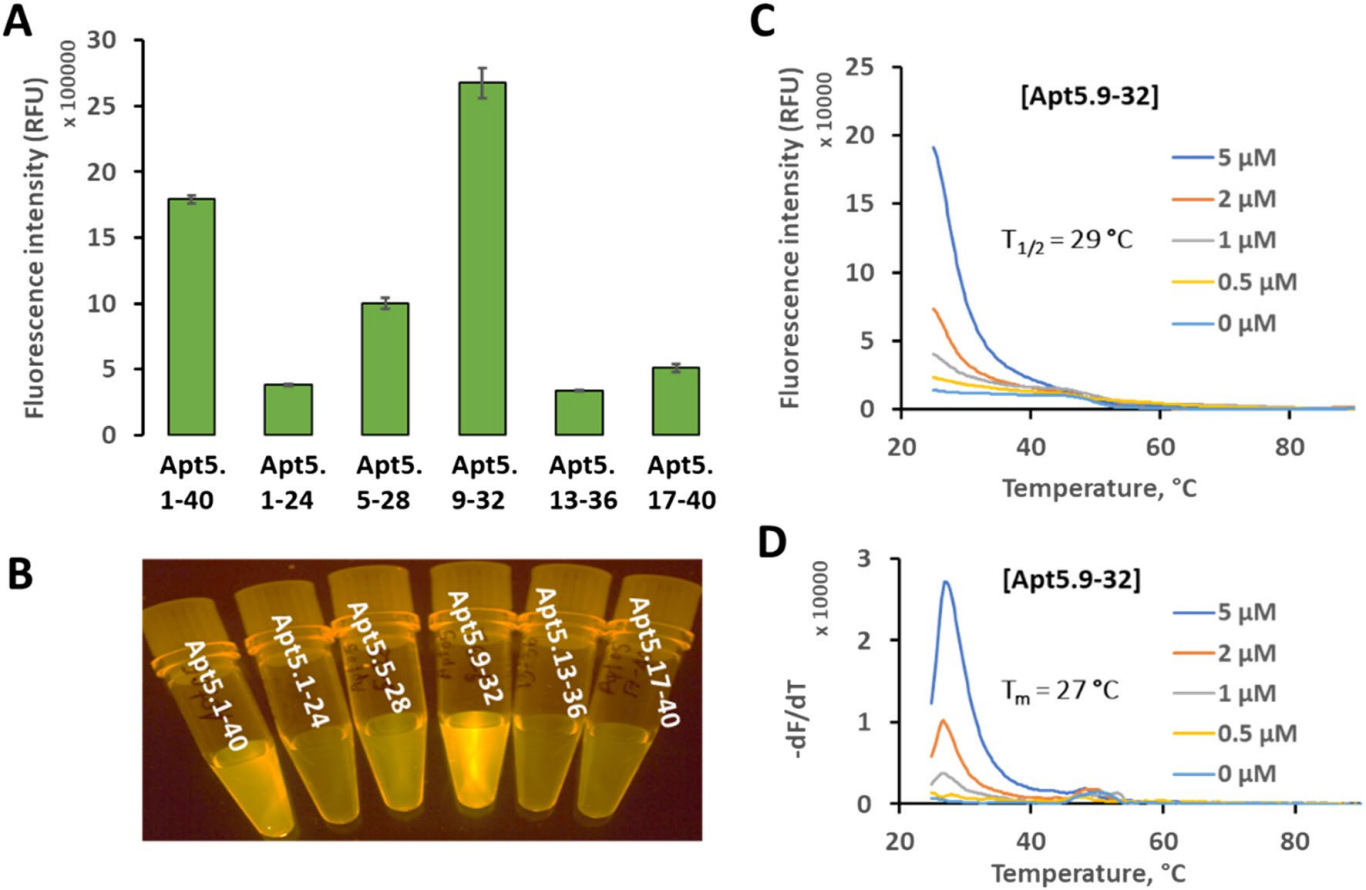
Aptamer minimization. (**A**) Fluorescence intensity of the best aptamer candidate Apt5 and five sub-sequences of Apt 5; 1-24, 5-28, 9-32, 13-36, and 17-40 at the concentration of 1 µM. (**B**) Image of fluorescence generated by Apt5 and five sub-sequences of Apt5. (**C**) Raw fluorescence melt curve of Apt5.9-32 (fluorescence as a function of temperature) analysis. T_1/2_ is defined as the temperature at which the fluorescence reached half its maximal value. (**D**) Negative 1st derivative plot of thermofluorometric analysis of Apt5.9-32 at the indicated concentrations from 0 to 5 µM.

We compared the ThT fluorescence enhancement of Apt5 and the five truncated versions. We generated secondary structure predictions for each with NUPACK. The NUPACK structural features did not correlate with fluorogenic activity. Of the five truncated aptamers, four were less active than the whole aptamer (including all versions with predicted secondary structure, see Fig. 3A). However, Apt5.9-32 showed increased fluorescence (despite showing no predicted structure in NUPACK) even relative to the full-length Apt5 (see Fig. 3A). NUPACK structure predictions are based on base-pairing interactions only and do not account for other structural elements, including quadruplexes or pseudoknots. NUPACK's predictions of an unfolded linear structure leave out some aspects of the real structures. Apt5.9-32 likely does have a preferred, folded conformation in solution but is not strongly base-paired. Apt5.9-32 is about 50% brighter than the original Apt5. When compared to the no-DNA control (blank, only ThT), Apt5.9-32 is about 90 × more fluorescent. Figure 3B also shows that Apt5.9-32 is visibly brighter than all other oligonucleotides tested. It may be that base-paired structures compete with the non-canonical structure that binds ThT. This lack of significant internal base-pairing is an advantage for our application. We require structures that can be easily denatured.

Although ThT is known to bind G-Quadruplexes^2^, ThT Apt5.9-32 is unlikely to be a strong G-quadruplex structure. It does not display known G-quadruplex repeat patterns such as (GGGNN)_4_. As noted in the introduction, its G4Hscore (0.833) is below 95% of sampled G-Quadruplexes^18^. The G4Hscore of Apt5, the full-length 40-mer aptamer, is 0.75. It is lower than the minimized aptamer, Apt5.9-32. Despite this fact, its fluorescence enhancement was significant. It is also noteworthy that Apt5.17-40 has a G4Hscore of 1.04, which indicates a strong possibility that it could adopt a G-quadruplex structure. NUPACK also predicts possible base-pairing structure as indicated in Fig. 2. Nonetheless, it is among the less fluorescent fragments.

We also performed a melt curve analysis of Apt5.9-32. We measured fluorescence as a function of increasing temperature using an Open qPCR instrument. We used 5 μM of ThT and four different concentrations of Apt5.9-32. Apt5.9-32 showed a T_1/2_ value at 29 °C (see Fig. 3C) and a peak in the negative first derivative (-dF/dT) at 27 °C (see Fig. 3D), which is a significantly lower temperature than a typical intramolecular G-quadruplex melting. For a detailed comparison, see Supplementary Figure S1. Potassium does not have any effect on the thermofluorimetry of Apt5.9-32. It has the same T_1/2_ and T_m_ values in the presence and absence of potassium (see Supplementary Figure S1 A,B). It has been reported that G-quadruplex structures are stabilized in solutions with high potassium concentration^12,36^. For comparison, a known G-quadruplex (denoted PW17Ext) has been used. It has a G4Hscore of 1.606^18^, which is typical for G-Quadruplexes. Likewise, potas sium shows a strong effect on PW17Ext. In the presence of potassium, PW17Ext made a more stable complex of ThT/PW17Ext. The presence of potassium increases both T_1/2_ and T_m_ values by 11 to 19 °C, respectively (see Supplementary Figure S1 C,D). While these results are circumstantial, they suggest that the interaction of ThT with Apt5.9-32 may be different from the known interaction of ThT with G-quadruplexes.

We also performed UV melt curve analysis of the Apt5.9-32. The aptamer may fold more efficiently at lower temperatures, but this is less relevant to our application. Because the polymerase is active at elevated temperatures, we performed UV melt curve analysis at room temperature and above. The results are shown in Supplementary Figure S2. The results were consistent with the fluorescence melt curve analysis of the Apt5.9-32.

We also performed CD spectroscopy of Apt5.9-32 with necessary controls, including a random ssDNA, dsDNA (Supplementary Figure S3). The negative peak of all the samples arise in the same wavelength position: 240 nm. However, the positive peak of the Apt5.9-32 is closer to that of the ssDNA and dsDNA. It is closer to the spectrum of ssDNA. The CD peak in Apt5.9-32 (~ 268 nm) was ~ 8 nm different from the characteristic G-quadruplex peak (260 nm). Also, the signal from the Apt5.9-32 is much less intense than a known G-quadruplex: PW17Ext or dsDNA control. We suspect the weak G-quadruplex-like peaks may arise from intermolecular interactions. At the recommended concentrations of oligonucleotides for the CD experiments (10 μM), the formation of intermolecular structures may be favored.

We also performed binding assays of Apt5.9-32 with ThT. We found that the average dissociation constant for Apt5.9-32 was 6 ± 2 µM of three individual measurements (see Supplementary Figure S4). It shows a moderate binding between the dye and aptamer. It is an advantage as the aptamer can be easily denatured in the presence of ThT while it still can act as a convenient reporter in the real-time DNA circuit monitoring.

Our aptamer has several advantages over the previous aptamer^19^ as a reporter of enzymatic amplification. It is shorter (24 vs. 33 nucleotides). It has much higher fluorescence when bound to ThT (see Supplementary Figure S5). Additionally, the minimized aptamer does not have a strong secondary structure, according to NUPACK, which is essential for designing strand displacement amplification (SDA) reactions. The lack of competing secondary structures is crucial for DNA polymerase to generate a new strand efficiently.

### Isothermal strand displacement amplification

We designed a target-triggered isothermal SDA reaction to detect miR-215. After optimizing the amplification, we determined that we could perform this SDA reaction at room temperature (or slightly above). It is a significant advantage: we did not require sophisticated temperature control. SDA is an enzymatic process that employed the activities of DNA polymerase and a nicking endonuclease. The microRNA miR-215 acted as a primer to initiate the SDA reaction.

We designed a DNA oligonucleotide to act as an SDA template. The Template-215 had three specific parts. The first part at the 3′ end is a sequence that was the reverse complement of the target miR-215. The second part of the SDA template was the reverse-complement of the nicking endonuclease recognition site (Nt.BstNBI). Finally, at the 5′ end of Template, the sequence is the reverse complement of the reporter, Apt5.9-32.

**Figure 4:**
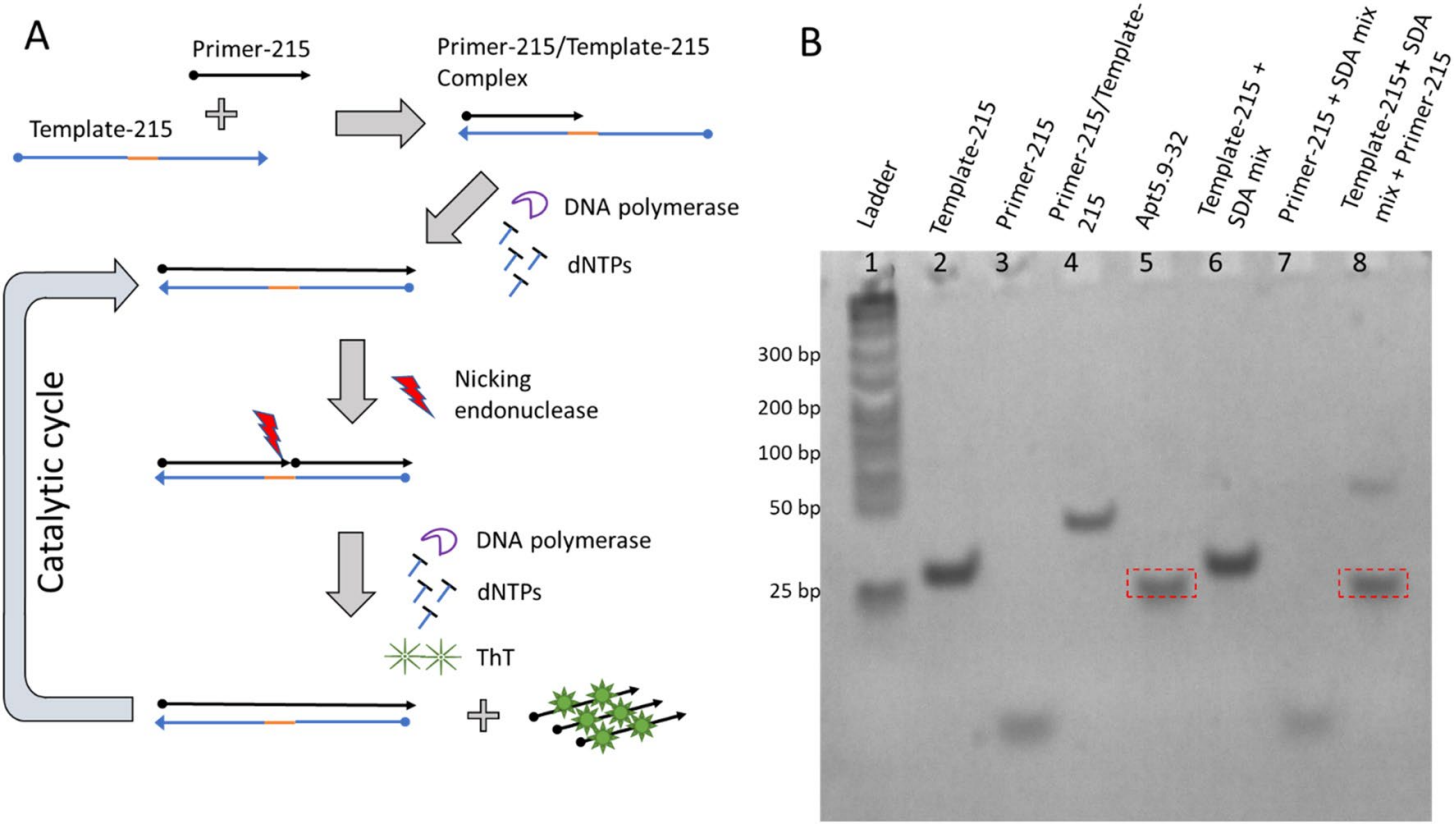
Strand displacement amplification (SDA) with ThT aptamer (Apt5.9-32) Reporter. (**A**) Schematic of strand displacement amplification with the ThT aptamer (Apt5.9-32) reporter system. (**B**) SDA product analysis by 12% PAGE. The red boxes indicate Apt5.9-32 bands.

SDA starts with the hybridization of miR-215 to the template strand (or a DNA oligonucleotide with the same sequence for testing purposes, denoted Primer-215). The polymerase binds to the 3′ end of the miR-215 and extends using dNTPs as fuel. Bst polymerase is promiscuous and highly processive and can extend either an RNA or DNA primer. As the new DNA is copied from the template strand, a nicking endonuclease recognition site is generated along with Apt5.9-32. (see Fig. 4A for the schematic of SDA). Once the primer strand extension is complete, the nicking endonuclease recognizes the specific nicking site and generates a single-stranded break or nick in the primer strand. It generates a new 3′ terminus on the primer strand. Bst polymerase can then extend again from this site. As it extends, it generates another copy of Apt5.9-32 and displaces the existing Apt5.9-32 into the solution with ThT. The catalytic cycle continues by nicking, extending, and releasing Apt5.9-32. The system generates many copies of Apt5.9-32 per input molecule. We estimate that every activated Template is copied ~ 30 times (based on the endpoint fluorescence and the relationship between aptamer and fluorescence in the binding curve). This gain in concentration is how we can achieve nanomolar detection limits for mir-215 despite the weak binding affinity between aptamer and dye.

We controlled for any direct interaction with miR-215 (Primer-215) and Apt5.9-32 with additional fluorescence measurements. We found no fluorescence generated by miR-215 in the presence of ThT. In addition to that, we also tested the same for Template and ThT and found no fluorescence. The results are shown in Supplementary Figure S6.

We analyzed the SDA system in optimized conditions using polyacrylamide gel electrophoresis (PAGE). Figure 4B is a negative image of the 12% PAGE gel under blue illumination. We have adjusted the contrast to highlight faint bands; the original, unadjusted image is included as Supplementary Figure S7. Lane 1 is a DNA Ladder. Lanes 2–4 are the SDA reaction components (Template-215, Primer-215, and Template-215/Primer-215 duplex, respectively, in Tris buffer). Lane 5 is synthetic Apt5.9-32 oligonucleotide, which the SDA reaction is designed to produce. Lane 6 and 7 contain Template-215 (no primer control) and Primer-215 (no template control) respectively in reaction conditions (i.e., including enzymes, dNTPs, Mg^2+^, and buffer). Lane 8 shows the full SDA reaction (i.e., including Primer-215 and Template-215). All mixtures were allowed to react for 50 min at room temperature before PAGE analysis. The key result is that the product (Apt5.9-32) is generated by the reaction only when Primer-215 and Template-215 are both present. The gel image shows that the SDA product band in lane 8 is aligned with the Apt5.9-32 band in lane 5 (indicated by boxes in Fig. 4B). We interpret the slower migrating band in lane 8, which is very faint, as the Template-215/Primer-215 complex in the SDA mix. This complex in Lane 8 migrated more slowly (compare to the Template-215/Primer-215 duplex in the Tris buffer in lane 4) due to the extension of the Primer-215 and the generation of fully double-stranded DNA.

### SDA characterization and optimization

We optimized the SDA reaction for reaction time as well as several parameters of the reaction composition. We determined the optimal endpoint by running the SDA reaction for 90 min while measuring fluorescence at every minute. It was found that negative control reactions were amplified after 60 min (see Supplementary Figure S8). We determined that the best endpoint for the reaction was 50 min.

Using the fluorescence at 50 min, we optimized temperature and the concentration of enzymes, dNTPs, MgSO_4_, and ThT dye. We measured the SDA performance based on three metrics: 1. the signal to background ratio (SBR), 2. the average rate of fluorescence increase, and 3. the ratio of specific to nonspecific product fluorescence. We focused on the SBR metric, but all three metrics were found to be highly consistent. The analysis in terms of the rate of fluorescence increase and the ratio of specific to nonspecific product fluorescence are presented in the supplementary information (see Supplementary Figure S9).

**Figure 5:**
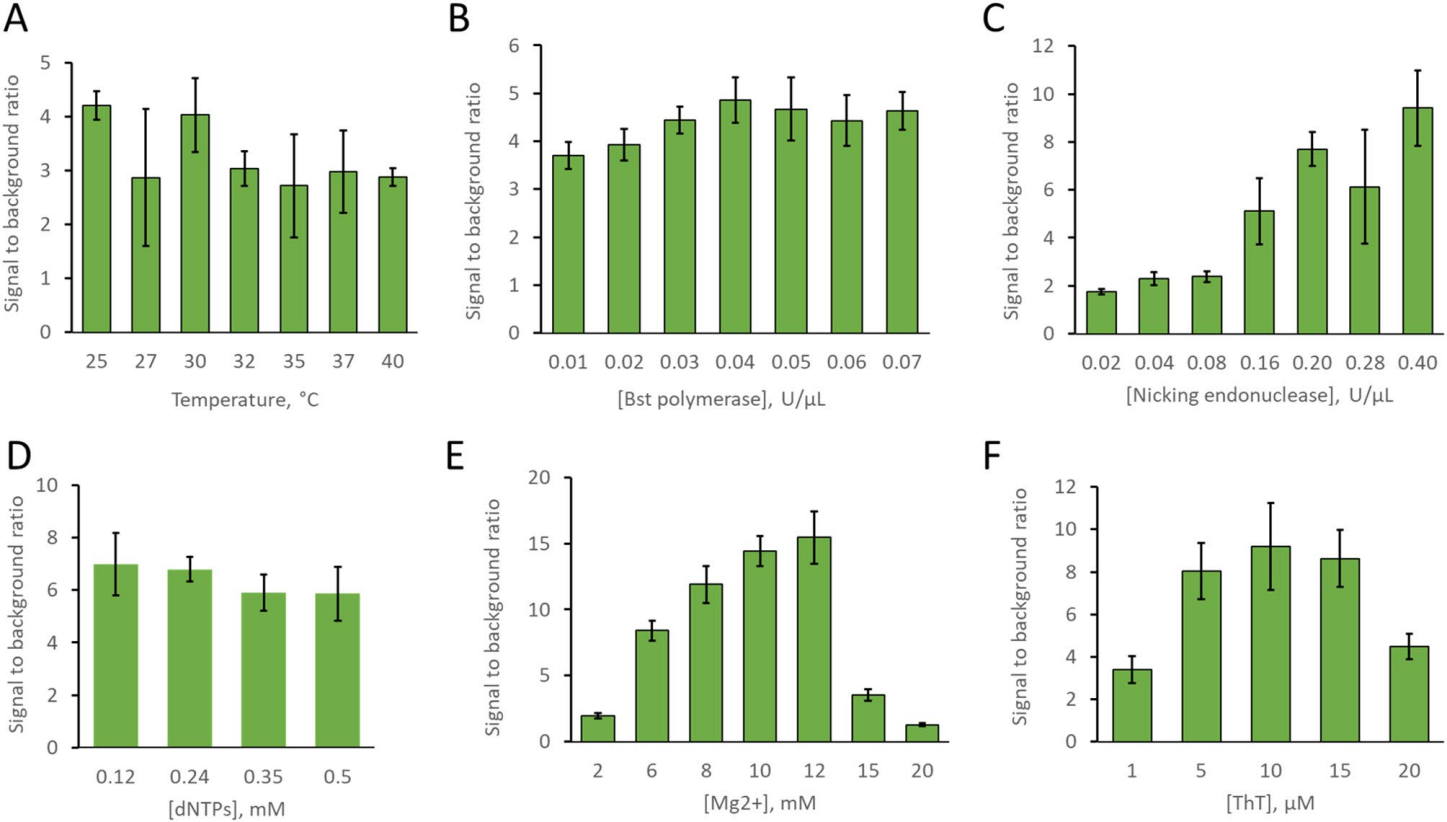
Optimization of six experimental parameters of the strand displacement amplification (SDA). Signal to background ratio (SBR) as a function of (**A**) Temperature, (**B**) Bst polymerase concentration, (**C**) Nicking endonuclease concentration, (**D**) dNTPs concentration, (**E**) Magnesium concentration, and (**F**) ThT concentration. Error bars represent the standard deviation of triplicate measurements in all cases.

The SBR is defined as the signal over the background. We define the signal as the fluorescence intensity of the primer plus template at 50 min. The background is defined as the intensity of the no-primer control at the same time point under the same conditions. Figure 5 shows the SBR as a function of changing each parameter.

First, we optimized the temperature of the SDA reaction. We sought to determine a reaction temperature that generated the highest SBR. We selected seven temperature points in the range of 25–40 °C (see Fig. 5A). Although we did not see a large SBR difference as a function of temperature, we noted that lower temperatures produce a slightly higher SBR. The consistent SBR over our temperature range also confirms that our SDA circuit design is a robust and flexible system able to operate at a wide temperature range. We selected 25 °C, room temperature, as the standard temperature for further experiments. This result is also consistent with the melt curve data in Fig. 3C,D. Apt5.9-32 has a fluorescence melting temperature of 27 °C. At this temperature, ThT/Apt5.9-32 lost fluorescence; this reduced the SBR of the SDA reaction.

We also optimized the concentration of the reaction components. Figure 5B–F shows the SBR as a function of the concentration of the reaction components. We interpret these results to mean that the SBR performance of the reaction was strongly dependent on nicking endonuclease concentration and magnesium ion concentration, suggesting that nick generation may be the limiting step in the catalytic cycle and that magnesium may enhance that reaction rate. The system was not strongly dependent on Bst polymerase, dNTPs concentration, or ThT concentration (within reasonable ranges). Based on these data, we standardized our conditions to 25 °C, 0.04 U/μL Bst polymerase, 0.2 U/μL Nt.BstNBI, 0.24 mM dNTPs (each nucleotide triphosphate), 10 mM Mg^2+^, and 8 μM ThT. When the performance was within the error of the optimal, we used the lower quantity. We conducted all experiments in triplicate.

### Strand displacement amplification (SDA) product confers reaction specificity

We were motivated to select an efficient aptamer against ThT to add specificity to the SDA reaction without the use of covalent labels. We tested whether the fluorescence response of the SDA system was specific to the aptamer in the presence of ThT dye. Nonspecific dyes are frequently used to monitor real-time DNA amplification reactions. However, the dyes respond to any DNA product, whether it is the intended product or some undesired side-product. We set out to show that the desired product of our SDA reaction, Apt5.9-32, would produce a signal in the presence of ThT, but aberrant reactions (that produce some other DNA) would not produce a signal.

**Figure 6:**
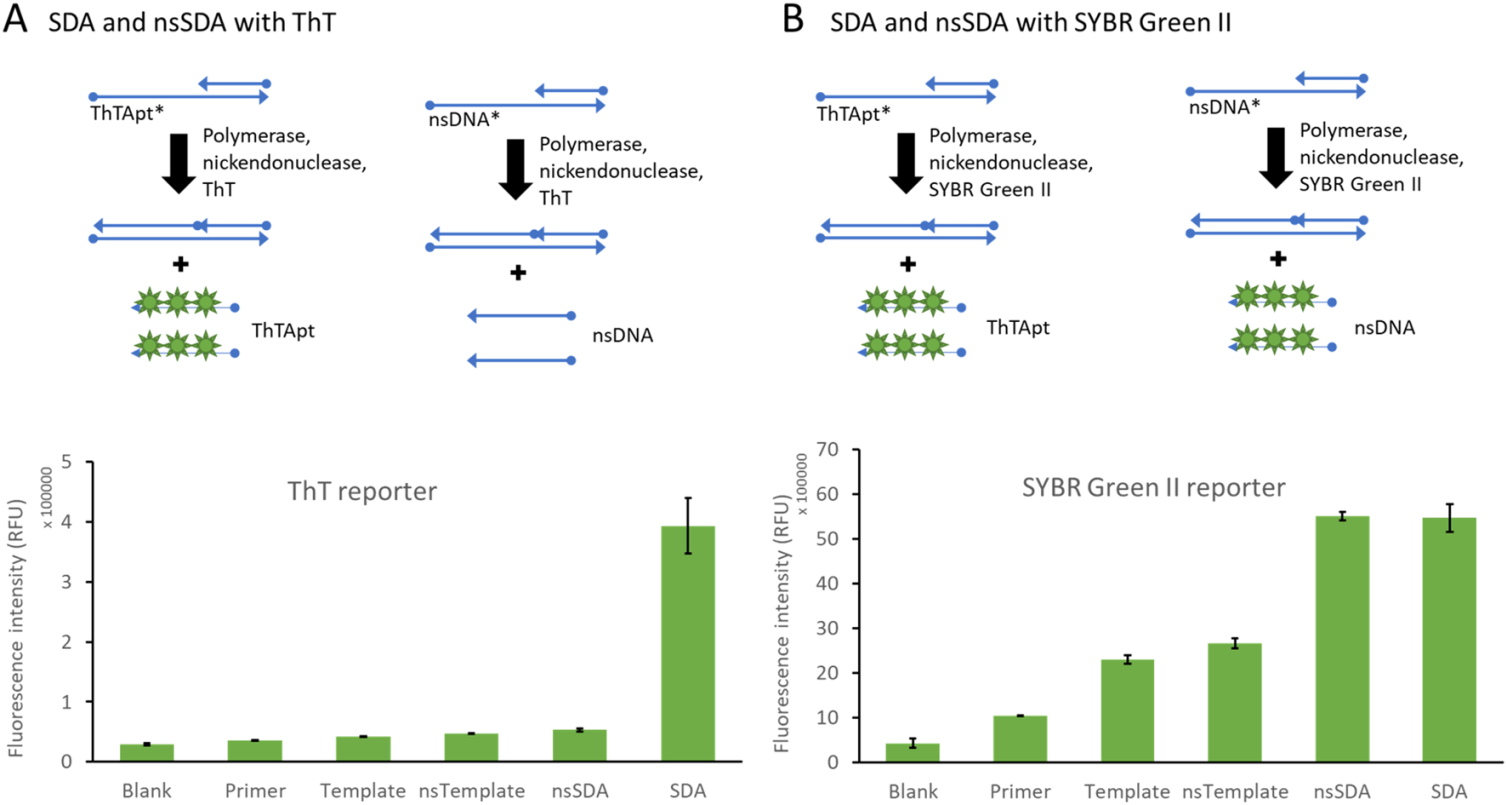
Strand displacement amplification (SDA) product specificity. (**A**) A schematic diagram (top) shows how SDA (aptamer product) and nsSDA (nonspecificSDA; SDA with the non-aptamer product) react in ThT. A bar graph shows fluorescence intensity (RFU) at 50 min with necessary controls (bottom). (**B**) A schematic diagram (top) shows how SDA and nsSDA react in SYBR Green II. A bar graph shows fluorescence intensity (RFU) at 50 min with necessary controls (bottom). Error bars represent the standard deviation of triplicate measurements in all cases.

We designed two different templates, denoted Template (to produce Apt5.9-32) and nsTemplate (nonspecific Template). The difference between the two sequences is that the Template contains the complementary sequence of Apt5.9-32 at the 5′ end, which generates Apt5.9-32 in the SDA system. The nsTemplate sequence contains a random sequence at the 5′ end, which generates nonspecific ssDNA in the system. We show that the SDA reaction (containing the Template oligonucleotide) lights up ThT present in the reaction. The nsSDA reaction (containing the nsTemplate oligonucleotide) does not light up the ThT in the reaction (see Fig. 6A).

This is a significant improvement over nonspecific dye like SYBR Green II. SYBR Green II binds to any single-stranded nucleic acid (DNA or RNA). SYBR Green II is fluorescent in the SDA reaction, the nsSDA reaction, and several negative controls (Fig. 6B). Both Apt5.9-32 and random ssDNA can generate signals. Although SYBR Green II was a brighter fluorophore under our conditions, ThT interacts specifically with the Apt5.9-32 product such that side reactions are suppressed. It confirms that our optimized SDA reaction generates an aptamer that we can detect against a background of other side products.

### Optimization of Template-215 concentration for the detection of miR-215

The SDA reaction is designed to produce Apt5.9-32 and become fluorescent when a miR-215 hybridizes to the Template-215. We optimized the Template-215 concentration to maximize the range and sensitivity of the SDA system. We varied Template-215 (the Template bearing the reverse complement of miR-215) at four different concentrations (25, 50, 100, 200 nM) (See Supplementary Figure S10A). At each concentration, we measured the rate of fluorescence increase as a function of Primer-215 (the DNA analogue of miR-215). The system responded best to Primer-215 when Template-215 concentrations were between 100 and 200 nM. Below this range, the strand displacement reaction is constrained by a lack of substrate and does not produce strong signals. However, higher template concentration tends to generate high nonspecific background. Figure S10B shows the time-course fluorescence data of the SDA system for various Primer-215 concentrations. The reaction is nearly linear with respect to time with an increasing slope as more Primer-215 is added. Figure S10C shows the linear relationship between endpoint fluorescence intensity and Primer-215 concentration in the SDA system. This data shows that the SDA system can act as a detector for Primer-215 with a linear range of 5–100 nM.

### Detection of miR-215

**Figure 7:**
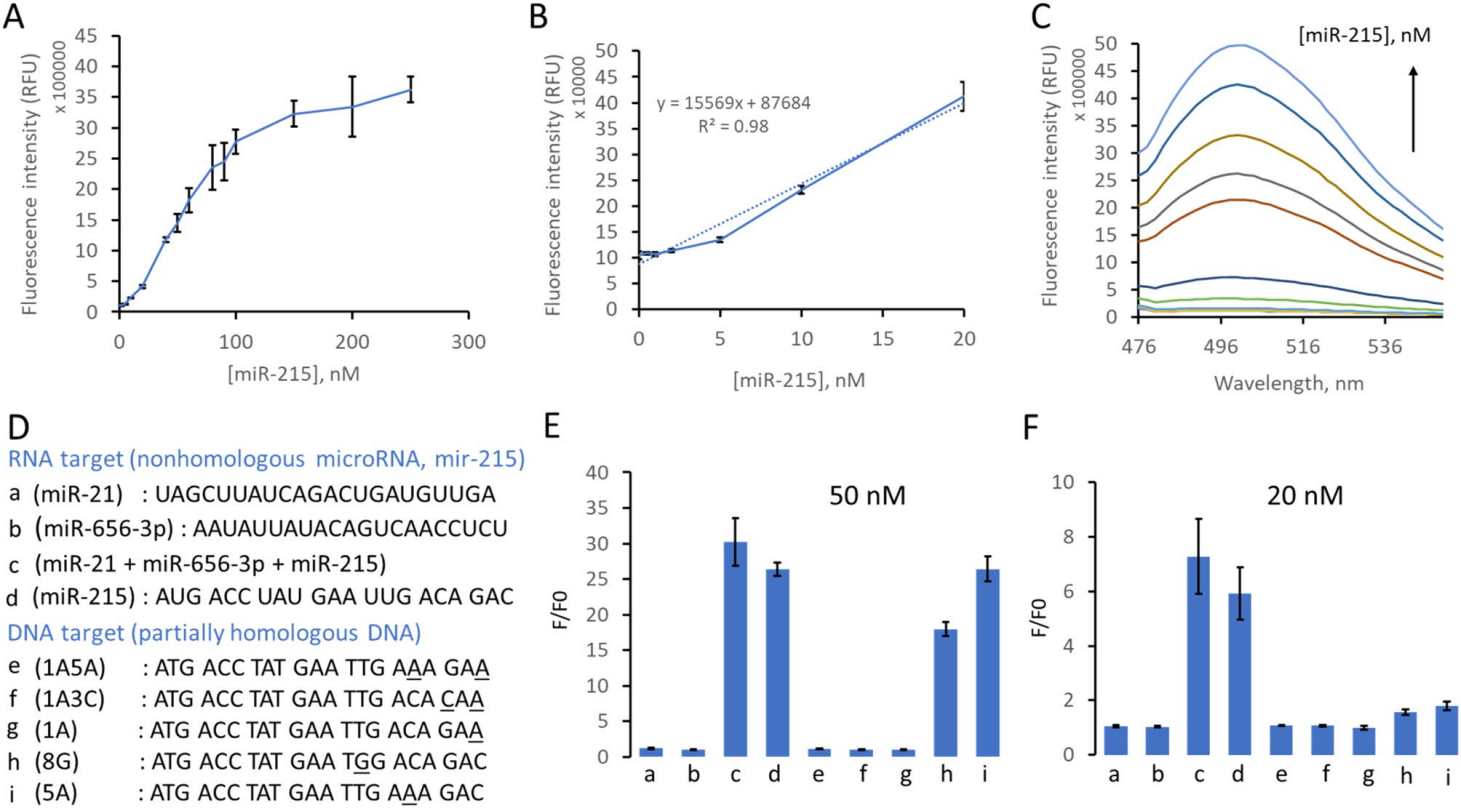
SDA Detection of miR-215. (**A**) Fluorescence intensity (RFU) of SDA reaction (with [Template-215] = 100 nM) as a function of miR-215 concentration (indicated concentrations from 0–250 nM). Fluorescence intensity was measured at 50 min. (**B**) Relationship between fluorescence intensity and miR-215 concentrations (indicated concentrations from 0–20 nM) in the SDA system (with [Template-215] = 100 nM). (**C**) Endpoint fluorescence spectra (at 50 min of the SDA reaction) containing different concentrations of miR-215 (0, 0.5, 1, 2, 5 10, 20, 40, 50, 60, 80, and 100 nM). (**D**) Sequences of target oligonucleotides (a-i) used for SDA specificity including microRNA targets (a–d) and partially homologous DNA targets (e–i). Underlined bases indicate one or two bases alteration with respect to the target miR-215 sequence. (**E**) SDA specificity with several target oligonucleotides (a−i), at 50 nM. (**F**) SDA specificity with several target oligonucleotides (a−i), at 20 nM. F/F0 is defined as the ratio of fluorescence intensity of SDA in the presence of target sequence (**F**) to the fluorescence intensity of SDA mix with no target (F0, reagent blank). Error bars represent the standard deviation of triplicate measurements in all cases.

We used our SDA system to detect miR-215 specifically. We used the optimized experimental conditions, including the optimal Template-215 concentration. Figure 7A shows excellent linearity and good sensitivity of the system. Figure 7B shows a linear relationship between fluorescence intensity at 510 nm and various miR-215 concentrations in the range of 0–20 nM. Figure 7C shows the fluorescence endpoint spectra (at time 50 min) of the SDA detection of miR-215. These results show that fluorescence intensity at 510 nm is the peak emission and increases dramatically as a function of the added miRNA. The regression equation of the linear range in Fig. 7B is shown with a correlation coefficient of 0.98. For target miR-215, the limit of detection of this label-free SDA system was calculated to be approximately 2.6 nM. This limit of detection is better than a similar study of ThT based SDA based miRNA detection^3^.

We investigated SDA specificity for the detection of miR-215. We used two nonhomologous RNA targets, miR-21 and miR-656-3p, and five designed partially homologous DNA targets. We designed DNA to contain one or two altered bases from the original sequence of miR-215 (see Fig. 7D). We named the partially homologous DNA sequences as 1A5A, 1A3C, 1A, 8G, and 5A, where the number indicates the position relative to the 3′ end and the letter is the new base. For example, 1A5A indicates the sequence where the 1st and 5th position from the 3′ end were altered to adenine. We found high specificity at 20 nM and reduced specificity at 50 nM (Fig. 7E). The detection was still specific for 3′ mismatched targets at the higher concentration but not specific for all internal mismatches. Bst DNA polymerase enzyme is known to be sensitive to 3′ mismatches^37,38^.

However, at 20 nM concentration, SDA specifically detects miR-215, and no other oligonucleotides were detected (Fig. 7F). The SDA shows better specificity at lower concentrations of target miRNAs. This is a more typical use case. The physiological concentrations of miRNAs are very low. SDA also successfully detected miR-215 when mixed with two other nonhomologous miRNA sequences used in the experiment. This shows that the SDA system can specifically detect miR-215 in the presence of other miRNAs.

We also investigated the potential of our system to detect miR-215 in more complex biological samples. We note that miRNA biomarkers have been measured in saliva^39,40^ and serum^41,42^. Therefore, we verified that the reaction functions in the context of those matrices. We made simulated biological samples by spiking the SDA mix to 10% of the relevant biofluid. Even in the presence of saliva or serum, miR-215 was successfully and specifically detected by the SDA system (Figure S11A). The fluorescence intensity in the presence of serum is lower than that of saliva, which is likely due to the presence of higher total protein concentration in the former. The overall result shows the excellent specificity of the SDA system in detecting miR-215 in a complex biological sample matrix (Figure S11B).

## Conclusion

We developed a new, light-up aptamer, Apt5.9-32, against ThT using modified GO-SELEX. This aptamer is brighter than the previously published work and is not predicted to have any base-pairing secondary structures. Isothermal nucleic acid-based amplifications are recognized for their simplicity in experiments and instruments in different bioanalytical applications^43^ in different platforms^44^. We developed a room temperature isothermal SDA reaction to generate a light-up aptamer. This reaction can successfully detect miR-215 (an important biological analyte). This label-free approach does not need covalently modified probes to report the activity of the system. Other single-stranded nucleic acid molecules (including other miRNAs) can be detected with this SDA system. The 3′ end of the SDA Template sequence simply needs to be redesigned to be complementary to the new 3′ end of the analyte. Critically, the generation of Apt5.9-32 by SDA adds a layer of specificity to the system without adding a second, modified probe to the reaction.

We demonstrated this reporter with the SDA reaction. While the nanomolar LOD of the ThT aptamer reporter with SDA is modest, it can be improved by adding a stronger amplification reaction. Other reactions built by stacking and combining the SDA reaction will also be amenable to detection by producing Apt5.9-32. It has the potential to be a universal reporter for other types of DNA amplification, such as EXPAR^45^, RCA^46,47^, SDA + RCA^22^, asymmetric PCR^48^, or EDA^8^. Input samples must be characterized to check that the sample matrix does not cause ThT to fluoresce; this can be determined with necessary controls, including no-template, no-primer, and no-enzyme controls. We hope to explore the broad applicability of this novel aptamer in future work.

## Supplementary Information

Supplementary Information.

## Data Availability

All data of this study are available from the corresponding author upon reasonable request.
