# Supplementary material for: Graphene oxide assisted light-up aptamer selection against Thioflavin T for label-free detection of microRNA
Source: Sci Rep. 2021 Feb 22;11:4291. doi: 10.1038/s41598-021-83640-z (PMC7900183; doi:10.1038/s41598-021-83640-z)
Supplement: Supplementary file 1 — Supplementary Information. [file 41598_2021_83640_MOESM1_ESM.pdf]

Supporting Information for: Graphene oxide assisted light-up aptamer selection against Thioflavin-T for label free detection of microRNA

Md Mamunul Islam, Victoria Ghielmetti, Peter B. Allen*

*E-mail: pballen@uidaho.edu

University of Idaho, Dept. of Chemistry, 001 Renfrew Hall, 875 Perimeter Dr, Moscow, ID 83844-2343

Table S1. Sequences of all oligonucleotides used in this study.

Name	Sequences
E7N40 pool	TGCTCCGACCTTAGTCTCTG[N40] GAACCGTGTAGCACAGCAGA
P1	TGCTCCGACCTTAGTCTCTG
P1-f	/56-FAM/TGCTCCGACCTTAGTCTCTG
P2	TCTGCTGTGCTACACGGTTC
P2-Acr	/5Acryd/TCTGCTGTGCTACACGGTTC
Apt1	TCGCGTGTGCAGAGGCGAGTAGGTGGGAGATCTGTCTGGG
Apt2	GACCGGAGGGGCATCAGCTGTCCGTGAGGTTGCCGCGAG
Apt3	GCACGTCCAGGACGGGGGAGCGGTGCTAGTGTCTGGCAGG
Apt4	GACCGGAGGGGCATCAGCTGTCCGTGAGGTTGCCGCGAGT
Apt5	GCGTAGATCGAGGCTATTAGGAGGTGGGATGCGTCAGGGC
Apt5.1-24	GCGTAGATCGAGGCTATTAGGAGG
Apt5.5-28	AGATCGAGGCTATTAGGAGGTGGG
Apt5.9-32	CGAGGCTATTAGGAGGTGGGATGC
Apt5.13-36	GCTATTAGGAGGTGGGATGCGTCA
Apt5.17-40	TTAGGAGGTGGGATGCGTCAGGGC
PW17Ext	GAGGAGGAGGAGGAGAGGGTAGGGCGGGTTGGG
B-Br V4	ATCGAATCCTCCTCCTCCTCGATACTCCTCCTCCTCGATA
G-Arm V4	TATCGAGGAGGAGGAGTATCGAGGAGGAGGAGGATTCGATTCATATGT
Template	GCATCCCACCTCCTAATAGCCTCGTTATGACTCAAAGATGGTACCTGCTTCTGAATT
Primer	AATTCAGAAGCAGGTACCATCTTT
nsTemplate	GTAGATATTCATACGGCTAATTGTTTATGACTCAAAGATGGTACCTGCTTCTGAATT
Template-215	GCATCCCACCTCCTAATAGCCTCGTTATGACTCTTTACTGTCTGTCAATTCATAGGTCAT
Primer-215	ATGACCTATGAATTGACAGAC
miR-215	AUGACCUAUGAAUUGACAGAC
miR-21	UAGCUUAUCAGACUGAUGUUGA
miR-656-3p	AAUAUUAUACAGUCAACCUCU
1A5A	ATG ACC TAT GAA TTG AAA GAA

1A3C	ATG ACC TAT GAA TTG ACA <u>C</u>A<u>A</u>
1A	ATG ACC TAT GAA TTG ACA GA<u>A</u>
8G	ATG ACC TAT GAA T<u>G</u>G ACA GAC
5A	ATG ACC TAT GAA TTG A<u>A</u>A GAC
ThT.2-2	GACGACGACACAGGATTAATCTTATTAGTCGTC

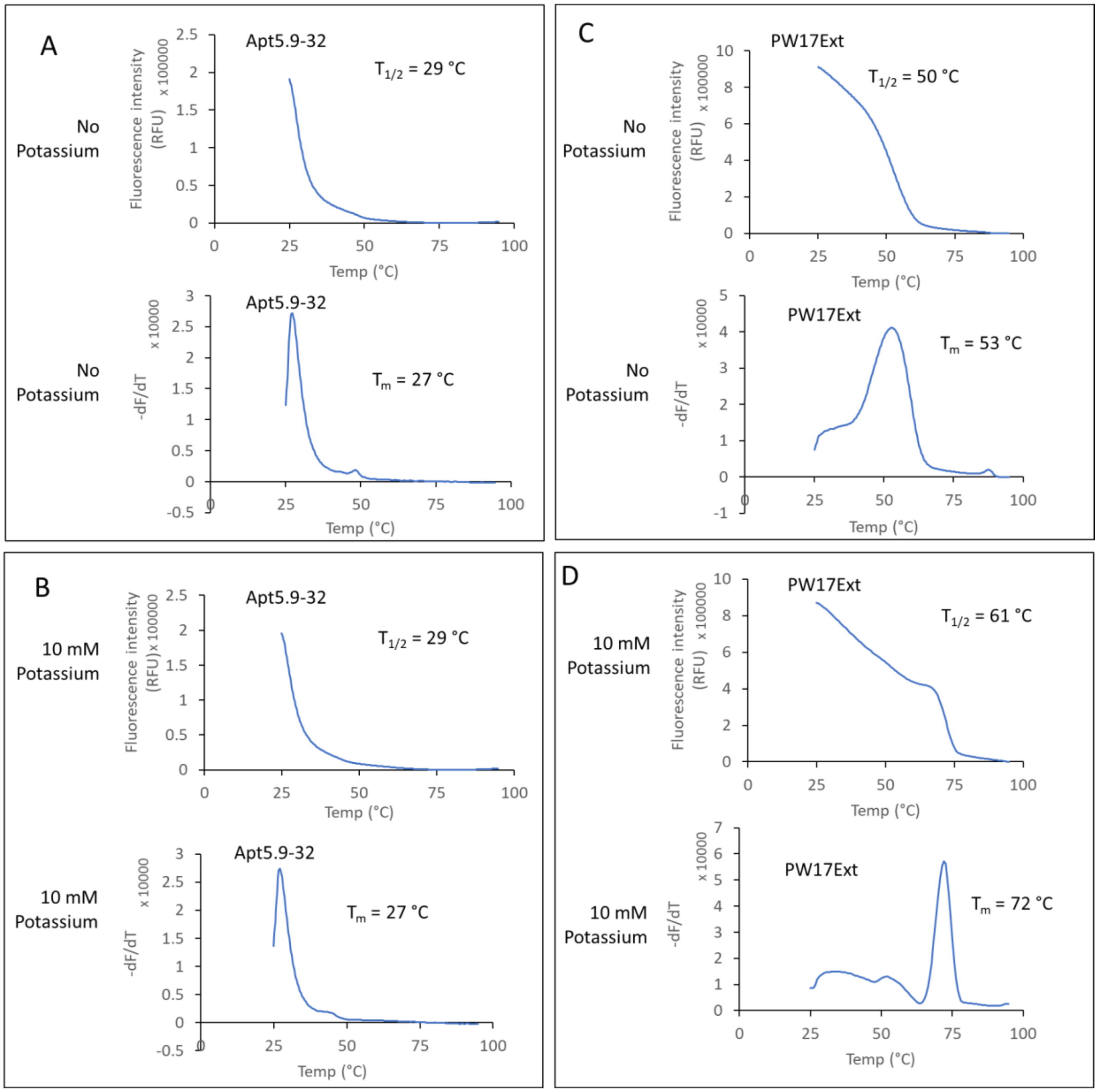

Figure S1: *Fluorescence melt curve analysis of ThT interaction with Apt5.9-32. Top graphs show the fluorescence as a function of temperature. Bottom graphs show the negative first derivative of fluorescence as a function of temperature. (A) Graphs show triplicate melt curves of Apt5.9-32 in the absence of potassium. (B) Graphs show triplicate melt curves of Apt5.9-32 in the presence of 10 mM potassium. (C) Graphs show triplicate melt curves of PW17Ext in the absence of potassium and (D) Graphs show triplicate melt curves of PW17Ext in the presence of 10 mM potassium. In each case,* $T_{1/2}$ *is defined as the temperature at which the fluorescence reached half its maximal value.*

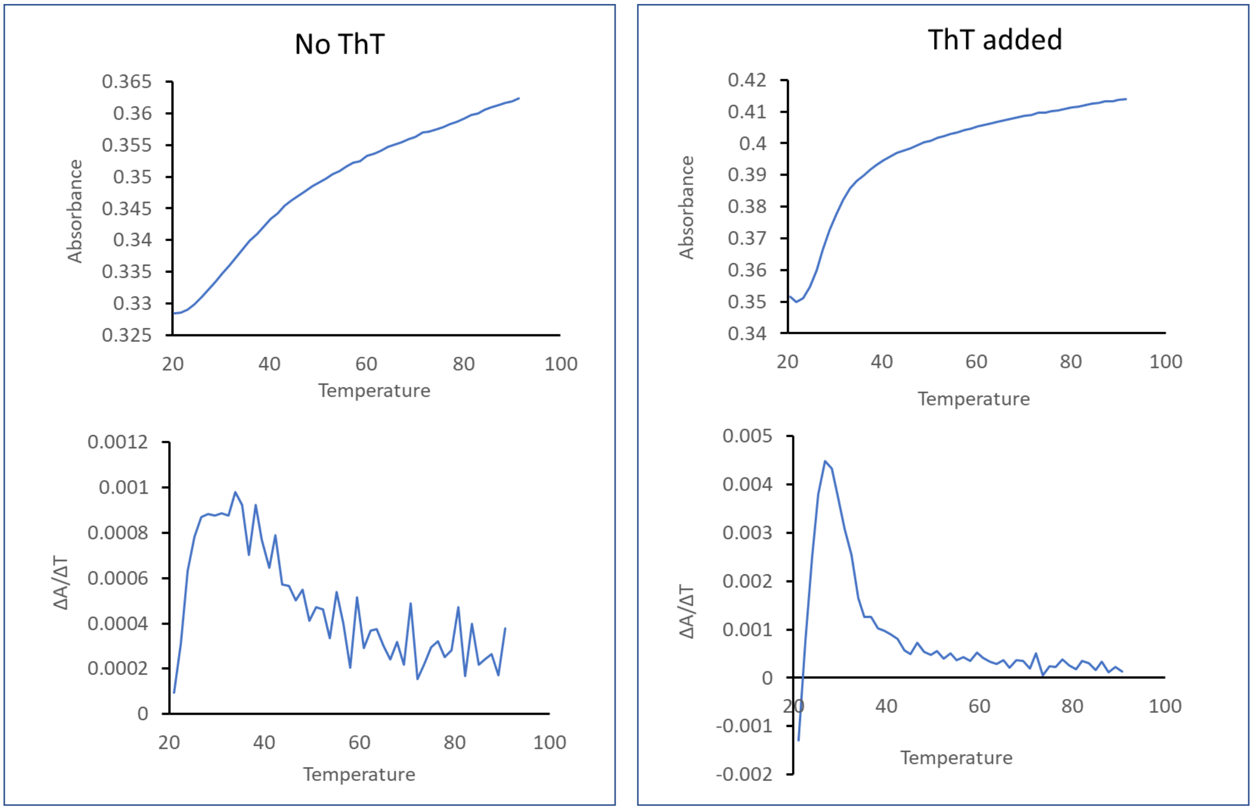

Figure S2: *Melt curve analysis of aptamer structure with UV absorbance at 260 nm. Top graphs show the absorbance as a function of temperature. Bottom graphs show the first derivative of absorbance as a function of temperature. Graphs show the duplicate absorbance as a function of temperature of the aptamer, Apt5.9-32 (at 1 μM) in two conditions: In selection buffer, no ThT(left) and in selection buffer, with 5 μM ThT (right).*

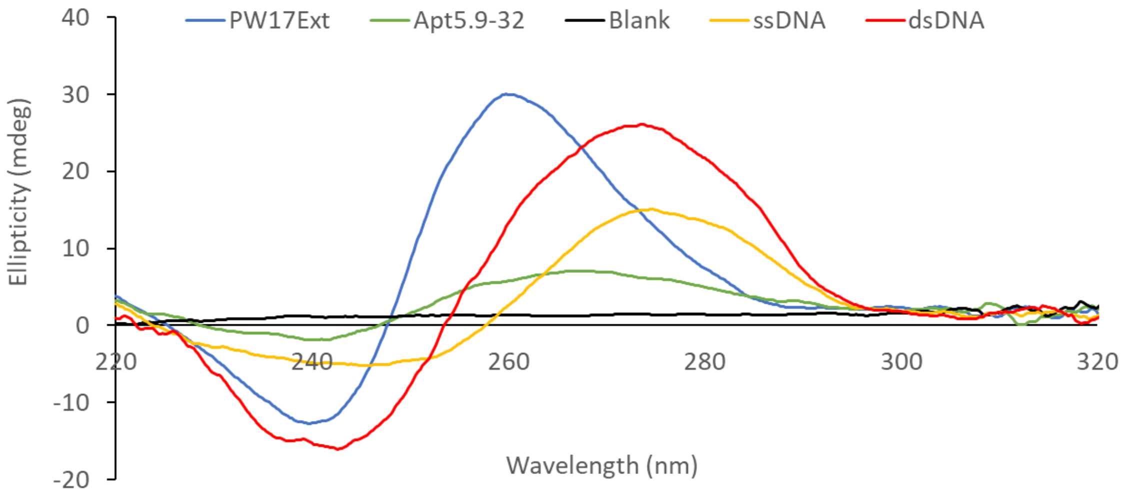

Figure S3: *CD spectra of the Apt5.9-32 with controls: PW17Ext (as a G4DNA), blank (buffer-no DNA), ssDNA (random), dsDNA (random). The experiment was performed at room temperature.*

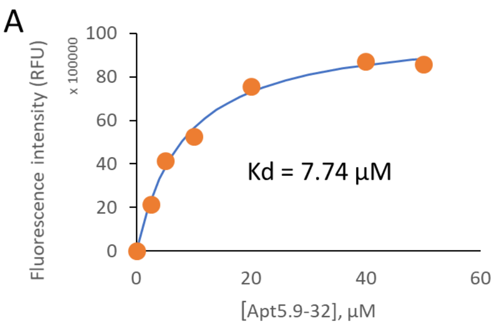

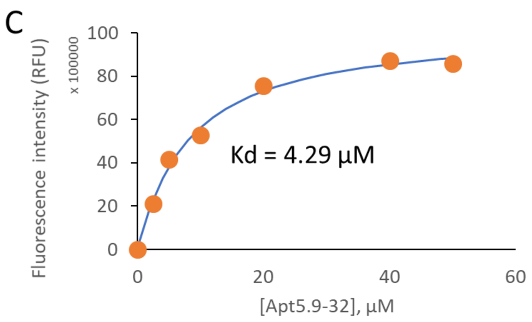

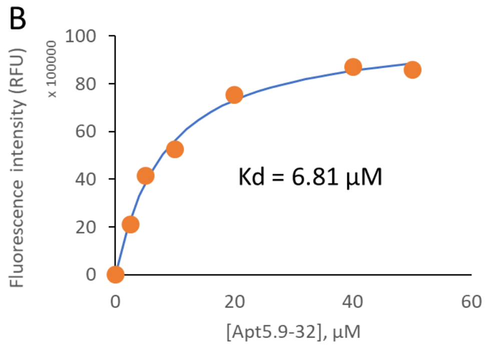

Figure S4: *Binding studies of Apt5.9-32 using Spectramax iD3 platereader. Graphs show Fluorescence intensity (RFU) as a function of (A) Apt5.9-32 concentration, trial1 (B) Apt5.9-32 concentration, trial2 C) Apt5.9-32 concentration, trial3*
(The blue line is the best fit binding isotherm used to determine dissociation constant (Kd) between Apt5.9-32 and ThT. Average Kd = 6.28 ± 1.78 µM. *The experiment was performed at room temperature.*

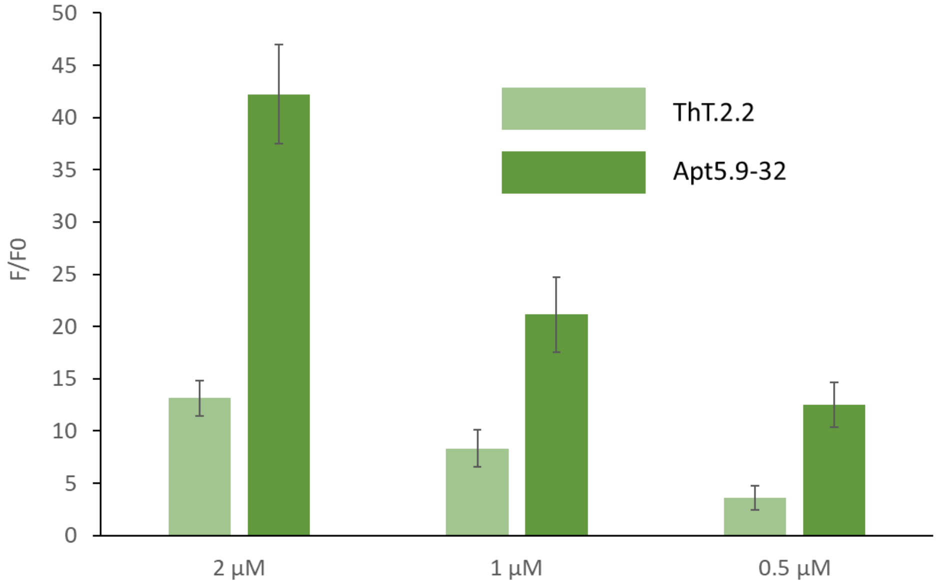

Figure S5: *Fluorescence enhancement of ThT comparison between Apt5.9-32(this work) and ThT.2-2 (Pei et al., Anal. Methods, 2016, 8, 8461) at several concentrations. (F0 is fluorescence of ThT only)*

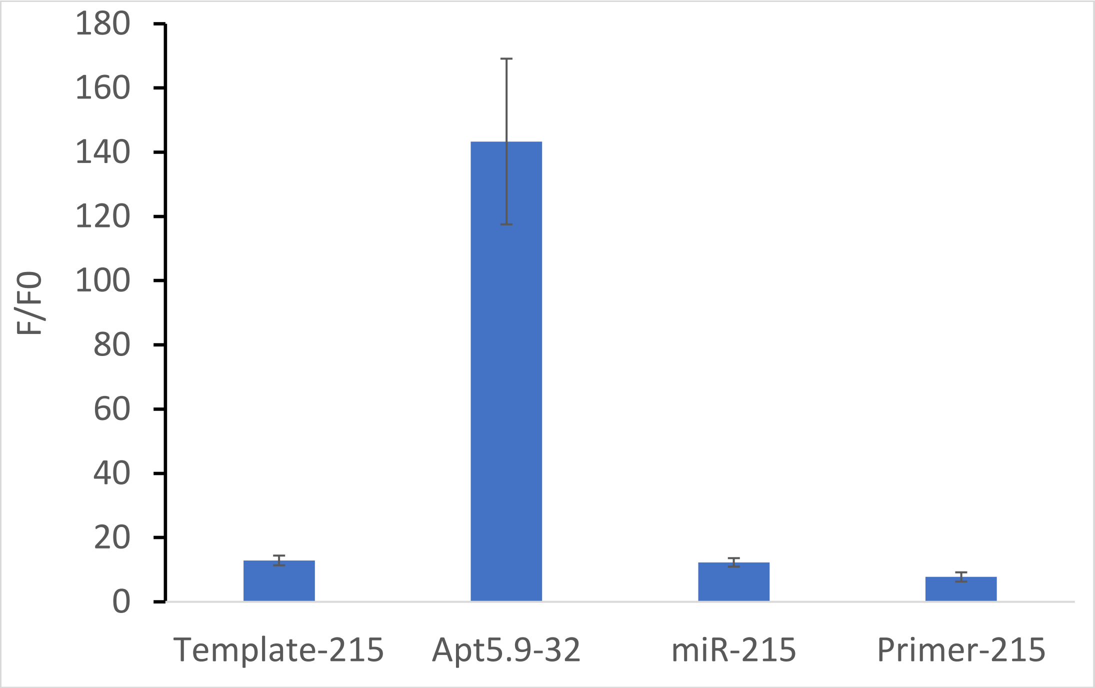

Figure S6: *Fluorescence enhancement of ThT (at 5 µM) by different oligonucleotides (at 1 µM). Comparison of fluorescence generated by Apt5.9-32, Template-215, miR-215, and Primer-215 (F0 is fluorescence of ThT only).*

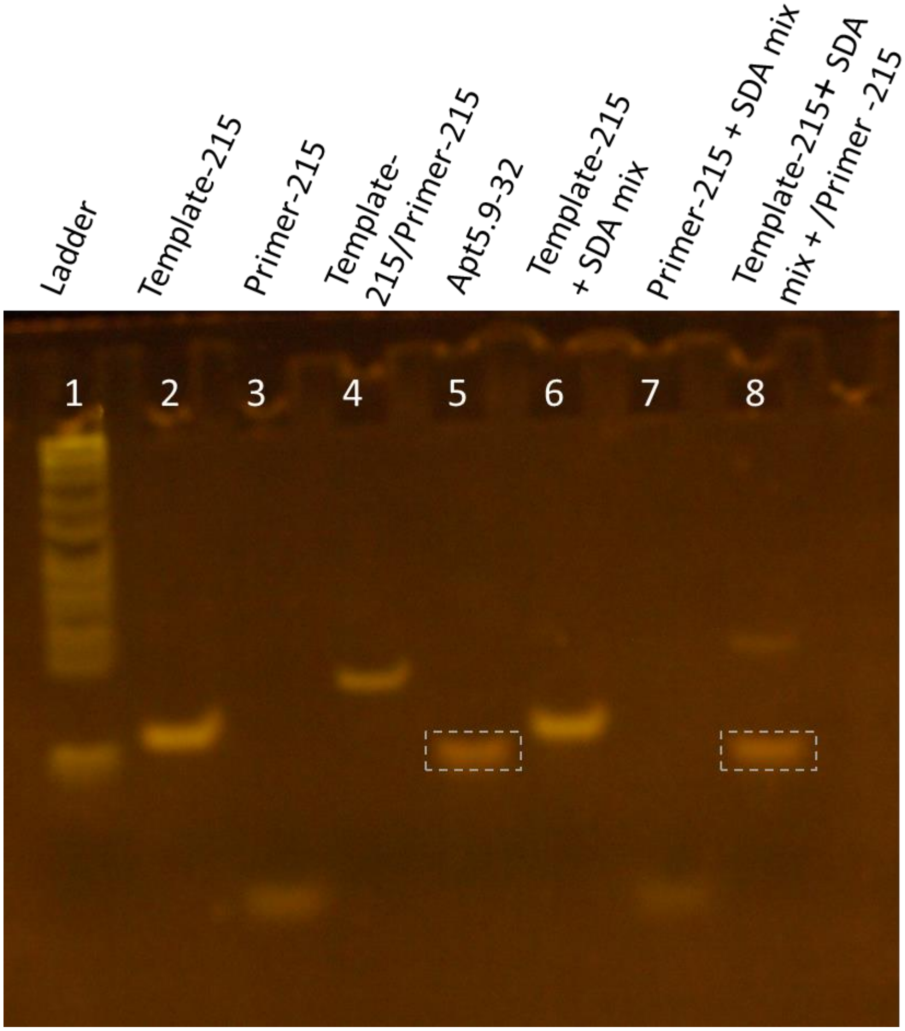

Figure S7: *SDA reaction analysis by 12 % native PAGE. SDA was compared against necessary controls: Template-215, Primer-215, Template-215/Primer-215 duplex and Apt5.9-32*

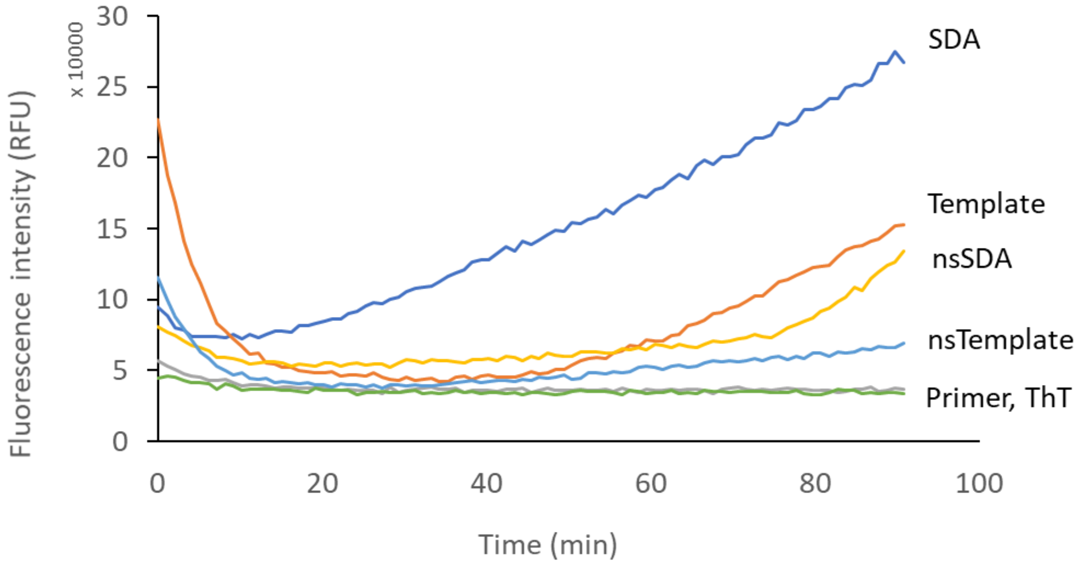

Figure S8: *SDA reaction time optimization. SDA was compared against necessary controls: Template, nsSDA, nsTemplate, Primer, and ThT*

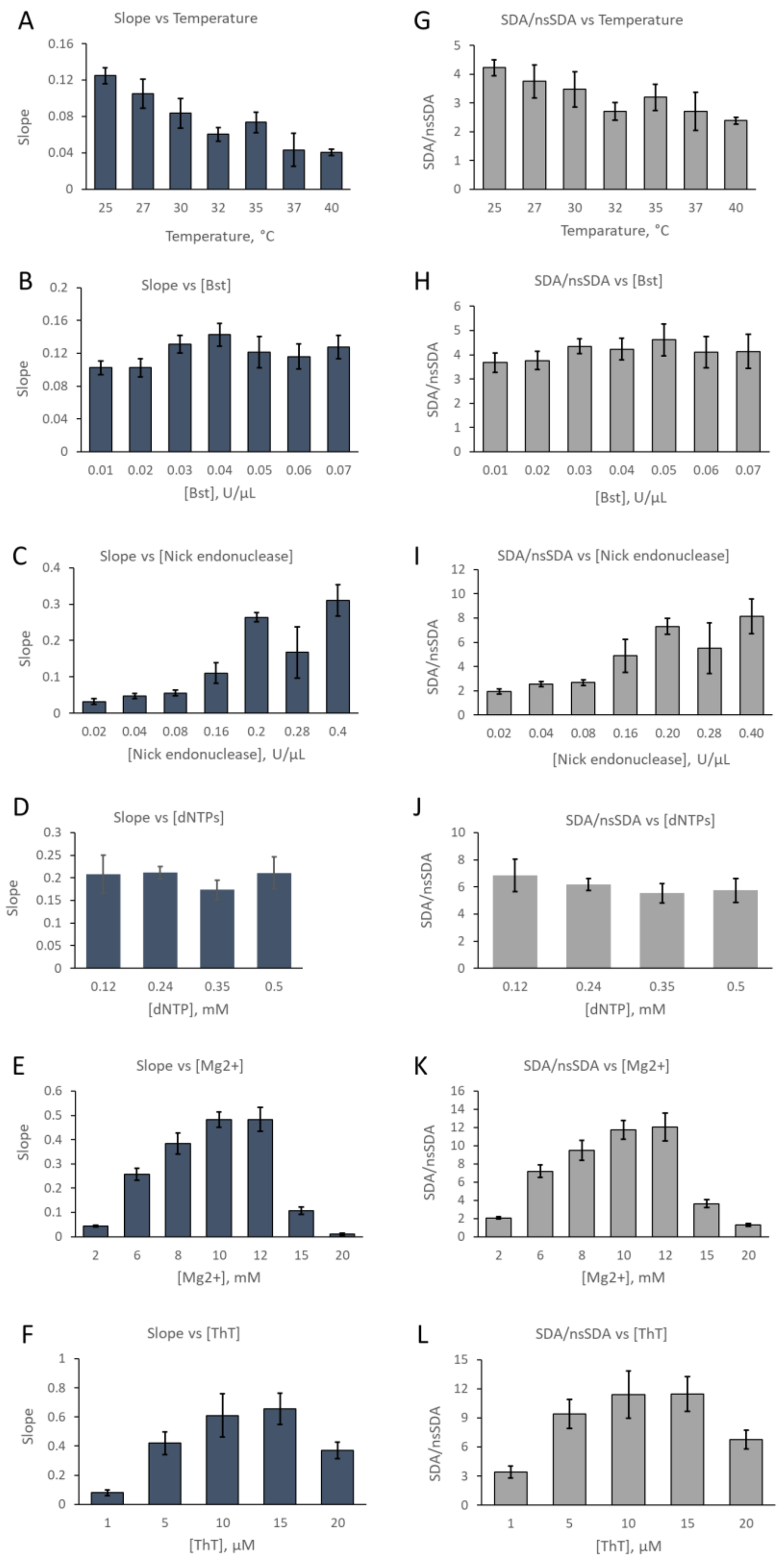

Figure S9: *Slope as a function of A) Temperature, B) Bst polymerase, C) Nick endo nuclease, D) dNTPs, E)* Mg^{2+}*, and F) ThT. SDA/nsSDA as a function of G) Temperature, H) Bst polymerase, I) Nick endo nuclease, J) dNTPs, K)* Mg^{2+}*, and L) ThT.*

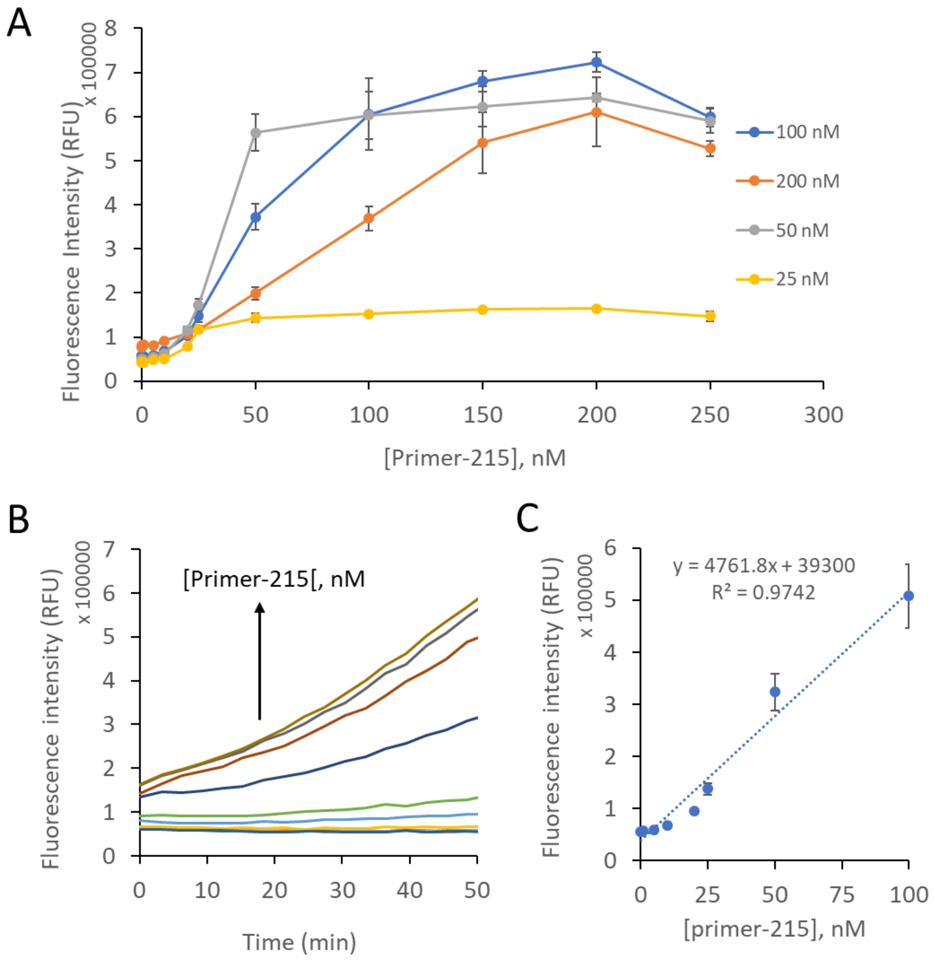

Figure S10: *Optimization of the Template-215 concentration. A) Fluorescence intensity of SDA reactions with four different concentrations of Template-215(100, 200, 50, and 25 nM) as a function of various Primer-215 concentration. Endpoint fluorescence intensity was collected at 50 min. B) Fluorescence-time curves as a function of different concentration of Primer-215 (0, 0.1, 1, 5, 10, 20, 25, 50, 100, 150, and 200 nM) where [Template-215] =100 nM. C) Linear relationship between fluorescence intensity and Primer-215 concentration in the SDA system where [Template-215] =100 nM (error bars represent the standard deviation of triplicate measurements in all cases).*

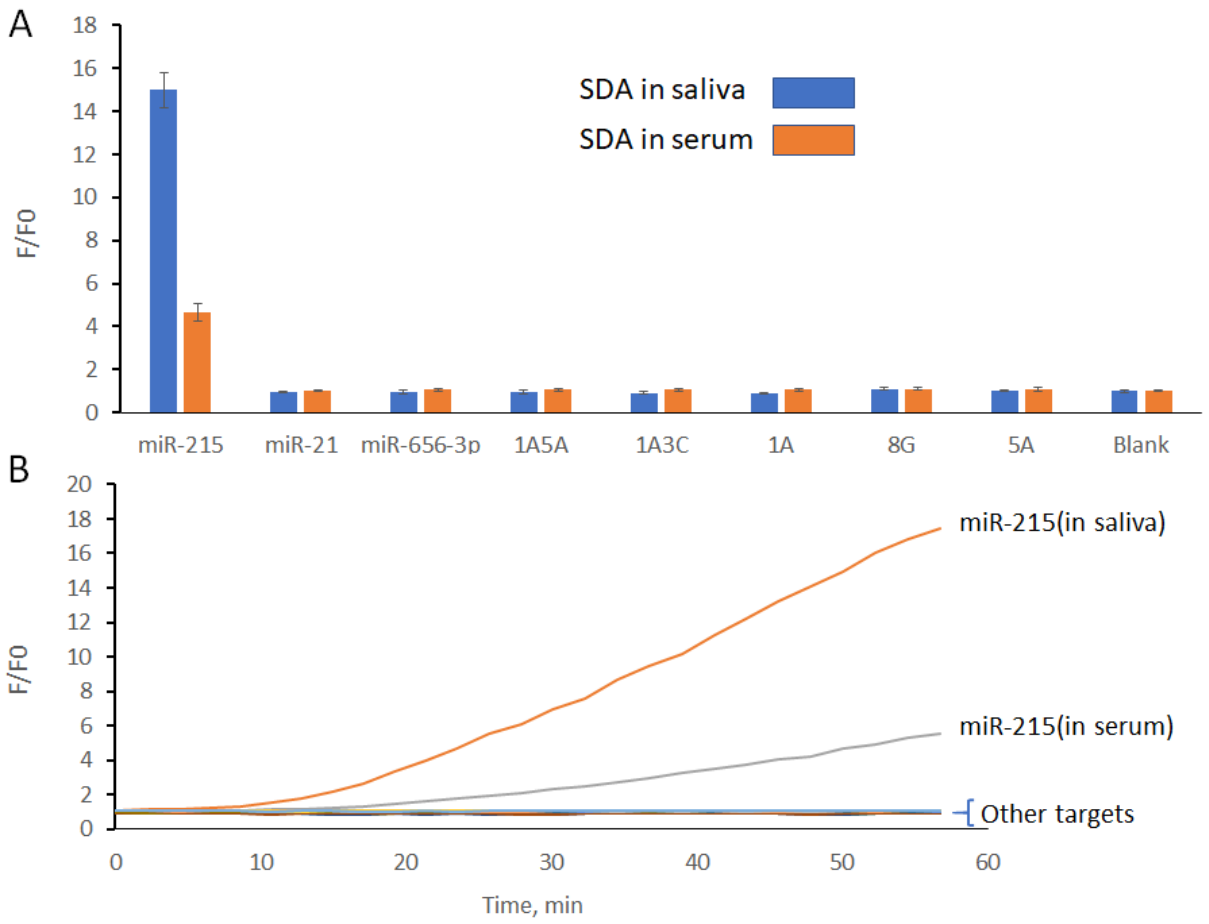

Figure S11: *SDA in two biofluids; saliva and serum. A) SDA comparison of 8 different oligonucleotides. Only the target miR-215 is detected both in saliva and serum. End point fluorescence intensity (F) was measured at 50 min. (error bars represent the standard deviation of triplicate measurements). B) Fluorescence-time curves as a function of 8 different oligonucleotides (triplicate measurements). Fluorescence intensity (F) from miR-215 is increased over the time in both saliva and serum sample (10% each). Other targets include miR-21, miR-656-3p, 1A5A, 1A3C, 1A, 8G, 5A, and blank in both saliva and serum samples. (F0 is fluorescence of SDA mix with no target)*
